# Activation of zebrafish Src family kinases by the prion protein is an amyloid-β-sensitive signal that prevents the endocytosis and degradation of E-cadherin/β-catenin complexes in vivo

**DOI:** 10.1186/s13024-016-0076-5

**Published:** 2016-02-09

**Authors:** Emily Sempou, Emiliano Biasini, Alejandro Pinzón-Olejua, David A. Harris, Edward Málaga-Trillo

**Affiliations:** Department of Biology, University of Konstanz, Constance, 78457 Germany; Present address: Department of Pediatrics, Yale University School of Medicine, New Haven, CT 06520 USA; Department of Biochemistry, Boston University School of Medicine, Boston, MA 02118 USA; Present address: Dulbecco Telethon Institute, Laboratory of Prions and Amyloids, Centre for Integrative Biology (CIBIO), University of Trento, 38123 Trento, Italy; Present address: Max PIanck Institute for Brain Research, Department of Synaptic Plasticity, 60438 Frankfurt/Main, Germany; Department of Biology, Universidad Peruana Cayetano Heredia, Lima 31, Perú

**Keywords:** Prion protein, Aβ oligomers, Zebrafish gastrulation, Neurodegeneration, Src family kinases, E-cadherin, β-catenin

## Abstract

**Background:**

Prions and amyloid-β (Aβ) oligomers trigger neurodegeneration by hijacking a poorly understood cellular signal mediated by the prion protein (PrP) at the plasma membrane. In early zebrafish embryos, PrP-1-dependent signals control cell-cell adhesion via a tyrosine phosphorylation-dependent mechanism.

**Results:**

Here we report that the Src family kinases (SFKs) Fyn and Yes act downstream of PrP-1 to prevent the endocytosis and degradation of E-cadherin/β-catenin adhesion complexes in vivo. Accordingly, knockdown of PrP-1 or Fyn/Yes cause similar zebrafish gastrulation phenotypes, whereas Fyn/Yes expression rescues the PrP-1 knockdown phenotype. We also show that zebrafish and mouse PrPs positively regulate the activity of Src kinases and that these have an unexpected positive effect on E-cadherin-mediated cell adhesion. Interestingly, while PrP knockdown impairs β-catenin adhesive function, PrP overexpression enhances it, thereby antagonizing its nuclear, wnt-related signaling activity and disturbing embryonic dorsoventral specification. The ability of mouse PrP to influence these events in zebrafish embryos requires its neuroprotective, polybasic N-terminus but not its neurotoxicity-associated central region. Remarkably, human Aβ oligomers up-regulate the PrP-1/SFK/E-cadherin/β-catenin pathway in zebrafish embryonic cells, mimicking a PrP gain-of-function scenario.

**Conclusions:**

Our gain- and loss-of-function experiments in zebrafish suggest that PrP and SFKs enhance the cell surface stability of embryonic adherens junctions via the same complex mechanism through which they over-activate neuroreceptors that trigger synaptic damage. The profound impact of this pathway on early zebrafish development makes these embryos an ideal model to study the cellular and molecular events affected by neurotoxic PrP mutations and ligands in vivo. In particular, our finding that human Aβ oligomers activate the zebrafish PrP/SFK/E-cadherin pathway opens the possibility of using fish embryos to rapidly screen for novel therapeutic targets and compounds against prion- and Alzheimer's-related neurodegeneration. Altogether, our data illustrate PrP-dependent signals relevant to embryonic development, neuronal physiology and neurological disease.

**Electronic supplementary material:**

The online version of this article (doi:10.1186/s13024-016-0076-5) contains supplementary material, which is available to authorized users.

## Background

Transmissible neurodegenerative disorders like Creutzfeldt-Jakob disease and bovine spongiform encephalopathy are commonly associated with the accumulation of misfolded prion protein (PrP^Sc^) in the brain. In fact, PrP^Sc^ oligomers can induce neuronal damage in vitro and in vivo [1, 2], and some of the cellular mechanisms involved have begun to emerge [3]. On the other hand, expression of the normally folded protein (PrP^C^) on the surface of neurons is necessary for PrP^Sc^ to effectively trigger neurodegeneration [4]. Moreover, some PrP mutations cause neurotoxicity in transgenic mice and familial prion disease patients even without inducing PrP^Sc^ formation [5, 6]. These observations strongly suggest that alterations in the function of PrP^C^ contribute to the early onset of neurodegeneration. This notion is further supported by the finding that PrP^C^ transduces neurotoxic signals from Aβ oligomers in Alzheimer’s patients [7, 8].

Uncovering mechanisms of PrP function in vivo remains a challenging task because no overt phenotypes are evident in PrP knockout mice, save for subtle abnormalities in olfactory physiology, neurogenesis, peripheral myelination and muscle regeneration [9–13]. Unfortunately, the biochemical basis of these defects and their connection to prion disease are yet to be established. On the other hand, in vitro studies have uncovered numerous putative roles for PrP^C^. Of special interest among these is the concept of PrP^C^ as a cell surface receptor and modulator of important signaling molecules like MAP kinases, PI3K/Akt, PKA, PKC and the SFK member Fyn [14, 15]. This non-receptor protein tyrosine kinase has been linked to prion [13, 16–18] and Alzheimer’s pathologies [19, 20] as well as to the synaptic impairment caused by binding of Aβ oligomers to PrP^C^ [7]. However, the physiological relevance of the PrP-Fyn interaction is unknown, as is the involvement of other SFK members in PrP-mediated events.

We formerly identified PrP as a positive regulator of cell-cell adhesion in vivo [21]. Morpholino knockdown of the zebrafish orthologue PrP-1 in early embryos led to the down-regulation of E-cadherin and the destabilization of adherens junctions (AJs). The progressive loss of tissue integrity in these morphant embryos resulted in gastrulation arrest due to their inability to carry out epiboly, an E-cadherin-dependent morphogenetic cell movement. Importantly, this lethal phenotype was partially rescued by expression of mouse PrP, highlighting the functional conservation among vertebrate PrPs. In the same study, we showed that PrP-1 engages in homophilic *trans*-interactions at cell-cell contacts and recruits Fyn to these sites. We therefore proposed that SFK-related signals mediate the effect of PrP-1 on embryonic cell adhesion [22].

Due to its anatomical simplicity, the zebrafish gastrula is ideally suited for biochemical and cell biological analyses upon genetic or pharmacological manipulations. Here, we used it to dissect the molecular mechanisms through which PrP and SFKs modulate embryonic AJ stability under physiological conditions. We also examined how this pathway is affected by PrP gain- and loss-of-function, by exposure to human Aβ oligomers, and by the expression of mouse PrP mutants with altered neurotoxic or neuroprotective properties.

## Results

### PrP-1 regulates the turnover of selected AJ components

**Fig. 1:**
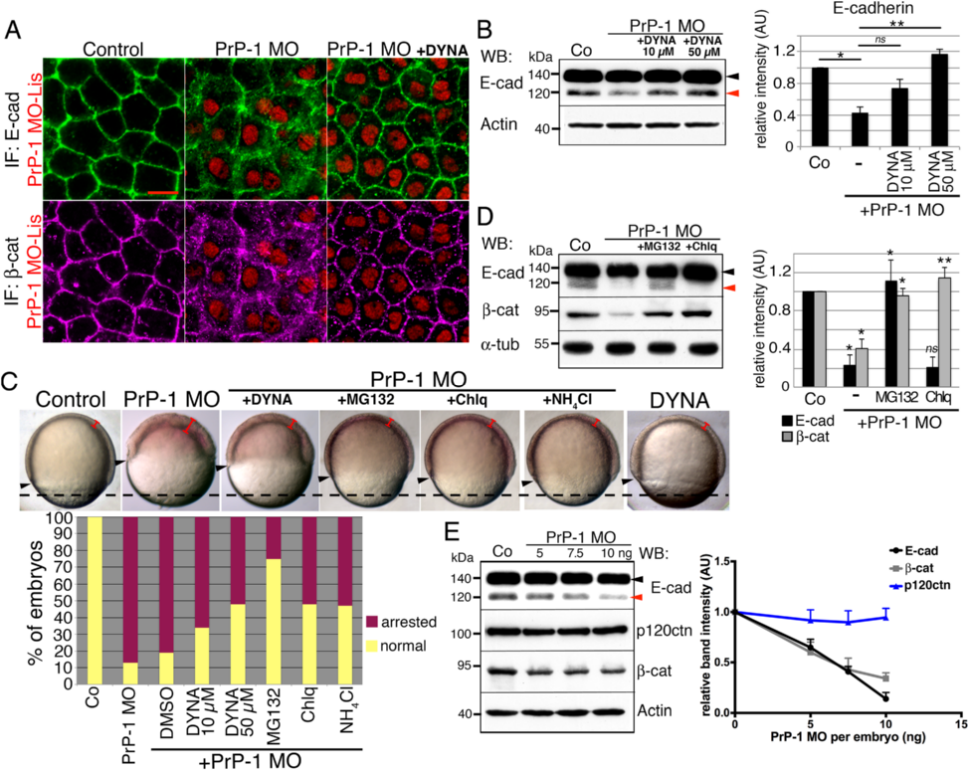
PrP-1 prevents the endocytosis and degradation of AJ components. **a**. E-cadherin (*green*) and β-catenin (*magenta*) localization in 6 hpf deep cells upon treatment with 10 μM Dynasore (DYNA); MO = lissamine-tagged PrP-1 morpholino (*red*); scale bar = 10 μm. **b** and **d**. Total levels of E-cadherin and β-catenin at 6 hpf upon treatment with Dynasore, MG-132 and Chloroquine (Chlq). **c**. Phenotypic analysis upon treatment with inhibitors. Top: progression of epiboly by 7.5 hpf is assessed by the downward extension of the embryonic margin (*arrowheads*) relative to the control (*dashed horizontal line*); brackets show the thickness of the blastoderm. Bottom: phenotypic quantification. Mean values are shown. **e**. AJ protein levels in 6 hpf embryos injected with increasing PrP-1 morpholino doses. WB = Western blot; IF = immunofluorescence. Red and black arrowheads in 1B, D and E indicate mature E-cadherin (120 kDa) and its more abundant precursor form (140 kDa), respectively. Densitometric analysis of Western blot bands is expressed in arbitrary units (AU) in B, D and E; average values of three independent experiments ± SEM are shown; statistical significance was assessed using unpaired, two-tailed *t-*tests; ns = not significant (*p* > 0.05), * = *p* ≤ 0.05, ** = *p* ≤ 0.01. In (**e**), E-cadherin and β-catenin levels were significantly reduced (*p* ≤ 0.05) at all three morpholino concentrations; p120ctn levels were not significantly changed at any morpholino dose (*p* > 0.05). See also Additional file 1: Figure S1

During gastrulation, the cell-surface expression of E-cadherin is dynamically regulated to promote tissue cohesiveness while allowing for coordinated cell movements [23]. In zebrafish embryos and mammalian cells, the 120 kDa cell-surface, mature E-cadherin protein is derived from the intracellular cleavage of a 140 kDa precursor polypeptide residing largely in intracellular vesicles [24, 25]. We reported that PrP-1 knockdown leads to depletion of mature E-cadherin but not its precursor form, suggesting that biosynthesis of the protein is normal but either its turnover or maturation are impaired [21]. To characterize this phenomenon, we first analyzed the effect of PrP-1 knockdown on E-cadherin internalization using immunofluorescence and Western blot. Treatment of morphant embryos with Dynasore, an inhibitor of clathrin- and dynamin-dependent endocytosis, restored the cell surface localization of E-cadherin by 6 hpf (Fig. 1a). In addition, Dynasore induced a concentration-dependent recovery in the levels of mature E-cadherin (Fig. 1b), indicating that its depletion in PrP-1 morphants is a consequence of increased endocytosis. Consistent with these molecular changes, Dynasore prompted a clear reduction -up to 40.23 %; *p* < 0.005- in the proportion of arrested/deformed PrP-1 knockdown embryos (Fig. 1c).

To determine the mechanisms of E-cadherin depletion in PrP-1 morphants, we treated them with MG-132 and chloroquine, inhibitors of proteasomal and lysosomal protein degradation, respectively. By 6 hpf, MG-132 caused a substantial recovery in the levels of mature E-cadherin, whereas chloroquine only enhanced the levels of its 140 kDa precursor form (Fig. 1d). Moreover, MG-132, chloroquine and ammonium chloride (another lysosomal inhibitor) improved the morphology of knockdown embryos (Fig. 1c), suggesting that both the mature and precursor forms of E-cadherin contribute to embryonic cell adhesion under these conditions. Notably, MG-132 -which restores the mature E-cadherin product- had the strongest rescuing effect of all inhibitors tested (71.26 % reduction in the proportion of arrested embryos, Fig. 1c) and restored E-cadherin localization (Additional file 1: Figure S1B). Together, these experiments indicate that in the absence of PrP-1, mature E-cadherin is increasingly endocytosed and degraded via a proteasome-dependent mechanism.

Binding of β-catenin to the cytoplasmic tail of E-cadherin helps anchor AJs to the actin cytoskeleton [26]. When detached from E-cadherin, free β-catenin accumulates in the cytosol and is rapidly degraded unless induced to activate gene transcription in the nucleus [27]. Given the strong cytosolic localization of β-catenin in PrP-1 morphant gastrulae (Fig. 1a), we examined the stability of this protein pool. Western blot and immunofluorescence analyses confirmed that β-catenin is internalized and strongly down-regulated in these embryos, and that this effect can be reverted by MG-132 or chloroquine (Fig. 1d and Additional file 1: Figure S1B). Interestingly, although β-catenin is not a direct target of the endocytic machinery, Dynasore treatment also restored its cell surface localization (Fig. 1a). Because β-catenin associates to the plasma membrane only indirectly via E-cadherin, this result implies that its internalization and degradation in PrP-1 morphants are secondary effects of E-cadherin endocytosis.

p120-catenin stabilizes E-cadherin at the cell surface by binding to its cytoplasmic tail and preventing its endocytosis [26]. Since p120-catenin depletion leads to the internalization and degradation of the entire AJ complexes, [28], we asked if its levels were reduced in PrP-1 morphants. Interestingly, Western blot revealed that while E-cadherin and β-catenin became increasingly degraded at higher PrP-1 morpholino doses, the levels of p120-catenin remained unaltered (Fig. 1e). Taken together, these data show that, in vivo, PrP-1 prevents the internalization and proteasomal/lysosomal degradation of E-cadherin/β-catenin complexes independently of p120-catenin.

### Fyn and Yes act downstream of PrP-1 to maintain embryonic AJ stability

**Fig. 2:**
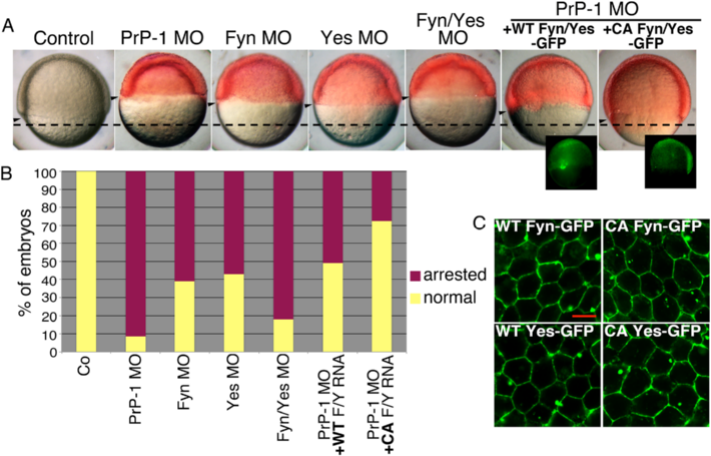
Fyn and Yes act downstream of PrP-1 during epiboly. **a**. 7.5 hpf embryos injected with different morpholinos or co-injected with PrP-1 morpholino and WT or CA Fyn/Yes EGFP mRNAs. Transmission and fluorescence images are shown merged (morpholinos in red). Expression of WT or CA Fyn/Yes-EGFP (green) is displayed separately on the lower right. Arrowheads and dashed horizontal line as in Fig. 1c. **b**. Corresponding quantification of embryonic phenotypes at 7.5 hpf. Mean values are shown. **c**. Expression pattern of WT or CA Fyn- and Yes-EGFP in deep cells of 7.5 hpf PrP-1 morphants. Scale bar = 10 μM

**Fig. 3:**
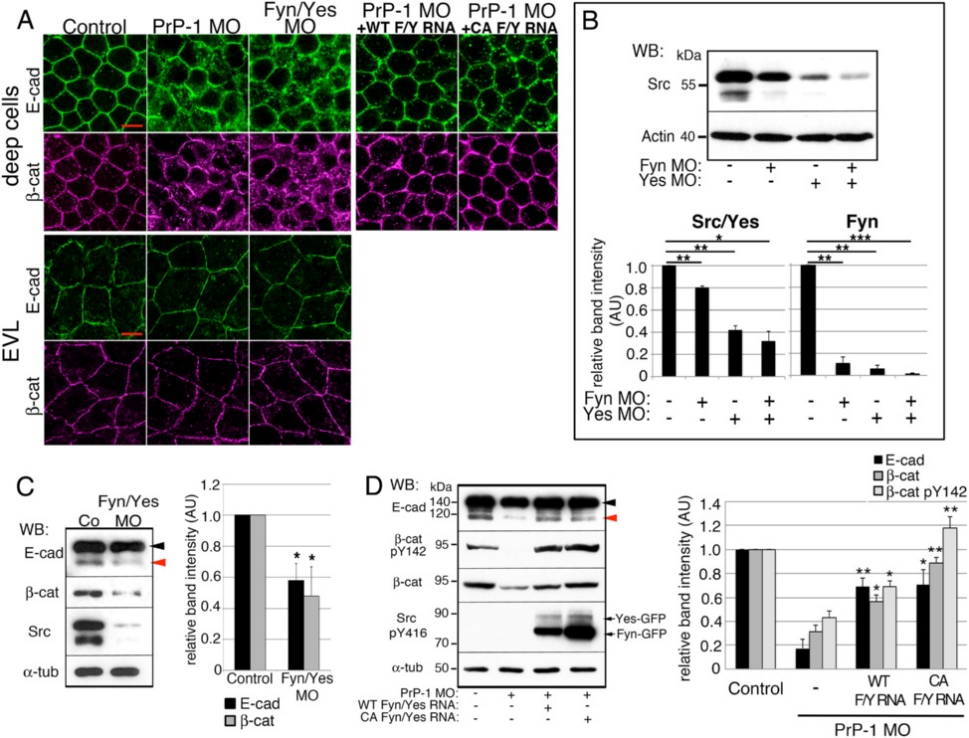
PrP-1 regulates AJs via Fyn and Yes. **a**. Localization of AJ components (immunofluorescence) in deep (*top*) and EVL cells (*bottom*) of 6 hpf PrP-1, Fyn/Yes morphants or PrP-1 morphants co-injected with Fyn/Yes mRNAs. Scale bars = 10 μM. See also Additional file 1: Figure S2. **b**. Reduction in Src/Yes (65 kDa) and Fyn (50 kDa) total levels in 6 hpf Fyn and Yes single or double knockdowns. **c**, **d**. Levels of AJ components in 6 hpf embryo lysates. Activated WT or CA Fyn- and Yes-EGFP were detected with an anti-phospho-Y416 Src antibody. E-cadherin arrowheads as in Fig. 1. WB = Western blot. Densitometric analysis of Western blot bands is expressed in arbitrary units (AU) in **b**, **c** and **d**; average values of three independent experiments ± SEM are shown; statistical significance was assessed using unpaired, two-tailed *t-*tests; ns = not significant (*p* > 0.05), * = *p* ≤ 0.05, ** = *p* ≤ 0.01, *** = *p* ≤ 0.001

PrP activates Fyn under diverse experimental conditions including cross-linking of PrP via antibodies as well as binding of PrP to other PrP molecules, NCAM or Aβ oligomers [7, 14, 21, 29]. In agreement with these data, we previously observed the accumulation of zebrafish embryonic Fyn at PrP-dependent cell-cell contacts [21]. On the other hand, genetic or chemical inactivation of Fyn and the related SFK Yes have been shown to impair zebrafish gastrulation [30–32]. Given the common involvement of SFKs in PrP signaling, zebrafish gastrulation, and the modulation of cadherin-based cell adhesion [33], we examined whether Fyn and Yes jointly mediate the effect of PrP-1 on AJs in vivo. First, we assessed the extent of the similarities between SFK and PrP loss-of-function phenotypes. At 6 hpf, *Fyn* and *Yes* single morphants showed epibolic arrest and deformed blastoderms (55–60 % of injected embryos), clearly resembling the PrP-1 morphant phenotype (Fig. 2a and b). The combined *Fyn*/*Yes* knockdown produced a stronger effect than the single knockdowns (82 % of injected embryos, Fig. 2a and b), indicating functional synergy between the two kinases. The protein knockdowns were titrated and verified by Western blot using an anti-Src antibody against the conserved C-terminus of SFKs (Fig. 3b): morphant embryos showed a clear reduction in the 65 and 50 kDa bands representing zebrafish Src/Yes and Fyn, respectively [34] (Fig. 3b). In line with the phenotypic quantifications, SFK down-regulation was more pronounced upon combined Fyn/Yes knockdown. We then asked whether the morphological resemblance between SFK and PrP-1 morphants involved common defects in AJ function. In fact, intracellular accumulation of E-cadherin and β-catenin was evident in Fyn and Yes, single and double knockdown embryos (Fig. 3a and Additional file 1: Figure S2A). Furthermore, Fyn/Yes morphant embryos displayed reduced levels of mature E-cadherin and β-catenin at 6 hpf (Fig. 3c). Hence, the morphological and molecular convergence of Fyn/Yes and PrP-1 knockdown phenotypes suggests that these proteins act cooperatively to stabilize embryonic AJ components.

Because SFKs transduce extracellular signals from trans-membrane receptors and GPI-anchored molecules like PrP [35, 36], we verified whether zebrafish Fyn and Yes are downstream effectors of PrP-1 and could therefore rescue the PrP-1 knockdown phenotype. We tested for this genetic interaction by co-injecting zebrafish Fyn and Yes EGFP-tagged mRNAs into PrP-1 morphants. To assess if the rescues were directly related to SFK enzymatic activity, we used wild-type (WT) and constitutively active (CA) forms of Fyn and Yes, the latter exhibiting higher kinase activity due to mutation of the conserved negative regulatory site at tyrosine (Tyr) 527. After verifying the proper localization and expression levels of the SFK constructs (Figs. 2c and 3d), their rescuing ability was quantified by scoring embryonic phenotypes between 6 and 7.5 hpf (Fig. 2a and b). Co-injection of *Fyn/Yes* mRNAs at 75 pg/embryo significantly reduced the proportion of arrested/deformed morphant embryos (41 % reduction, *p* < 0.01). Notably, CA *Fyn/Yes* mRNAs produced a more efficient rescue of the PrP-1 phenotype (64 % reduction; *p* < 0.01) using less mRNA (6 pg/embryo) (Fig. 2b). The higher enzymatic activity of the CA constructs was confirmed by Western blot using phospho-specific antibodies against activated SFKs (phosphorylated at Tyr416) and their target residue on β-catenin (phosphorylated at Tyr142) [37]. These Western blots show that the depletion of phospho-β-catenin in PrP-1 morphants is reversed upon expression of increasingly active WT and CA Fyn/Yes (Fig. 3d). Finally, we verified if SFK expression induced the recovery of AJ components in PrP-1 morphant embryos. Indeed, exogenous Fyn and Yes restored the cell surface localization and levels of E-cadherin and β-catenin by 6 hpf (Fig. 3a and b). Altogether, these data indicate that Fyn and Yes act downstream of PrP-1 to promote AJ function via tyrosine phosphorylation.

### PrP-1 modulates embryonic SFK function

**Fig. 4:**
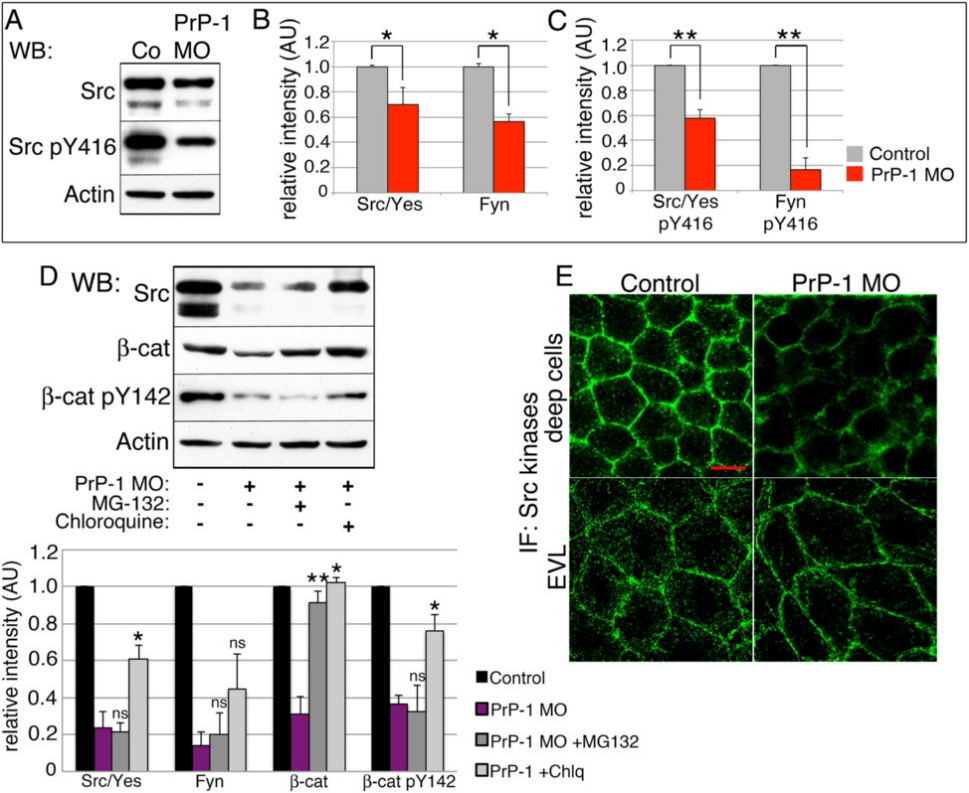
PrP-1 modulates SFK levels and activation. **a**-**c**. Levels of total and activated (phosphor-Y416) SFKs in 6 hpf embryos (**a**), and densitometric analysis of Western blot bands (**b** and **c**). Phospho-SFK values were normalized to those of total SFKs. Average values ± SEM of four independent experiments are shown. **d**. SFK levels in 6 hpf embryos upon PrP-1 knockdown and additional treatment with degradation inhibitors. **e**. SFK localization in deep (*top*) and EVL cells (*bottom*) of 6 hpf embryos. WB = Western blot; IF = immunofluorescence. Scale bar = 10 μM. Densitometric analysis of Western blot bands (**b**, **c** and **d**) is expressed in arbitrary units (AU); average values of four independent experiments ± SEM are shown; statistical significance was assessed using unpaired, two-tailed *t-*tests; ns = not significant (*p* > 0.05), * = *p* ≤ 0.05, ** = *p* ≤ 0.01. See also Additional file 1: Figure S3

Having identified Fyn and Yes as downstream effectors of PrP-1 in vivo, we next asked whether PrP modulates the levels and activity of SFKs. In fact, Western blots of 6 hpf embryos showed reduced levels of Src/Yes (65 kDa band, 29.77 % reduction; *p* < 0.05) and Fyn (50 kDa band, 43.36 % reduction; *p* < 0.05) upon PrP-1 knockdown (Fig. 4a and b). This effect, also evident in whole mount immunostainings (Fig. 4e), became more pronounced at higher morpholino doses (Additional file 1: Figure S3A) and could be partially rescued with chloroquine but not with MG-132 (Fig. 4d). Interestingly, PrP-1 knockdown and chloroquine also modified the levels of phospho-Tyr142-β-catenin, suggesting that PrP-1 effectively modulates SFK catalytic activity. To verify this, we measured the ratios of active and non-active SFKs in PrP-1 morphants. Using phospho-specific antibodies, we found a significant decline in the ratios of active (phosphorylated at Tyr416) vs. total Src/Yes and Fyn (44.28 % and 83.29 % reduction, respectively; *p* < 0.01) upon PrP-1 knockdown (Fig. 4c). Notably, no significant changes were observed in the ratios of inactive (phosphorylated at Tyr527) vs. total Src/Yes and Fyn (Additional file 1: Figure S3B), indicating that PrP-1 preferentially stabilizes the levels of activated SFKs. Accordingly, overall tyrosine phosphorylation was reduced in the deep cells of PrP-1 morphants (Additional file 1: Figure S3C). Therefore, our experiments show that PrP-1 positively modulates the levels and activity of SFKs in the gastrula, partly by preventing their lysosomal degradation.

### Embryonic PrP gain-of-function phenotype

**Fig. 5:**
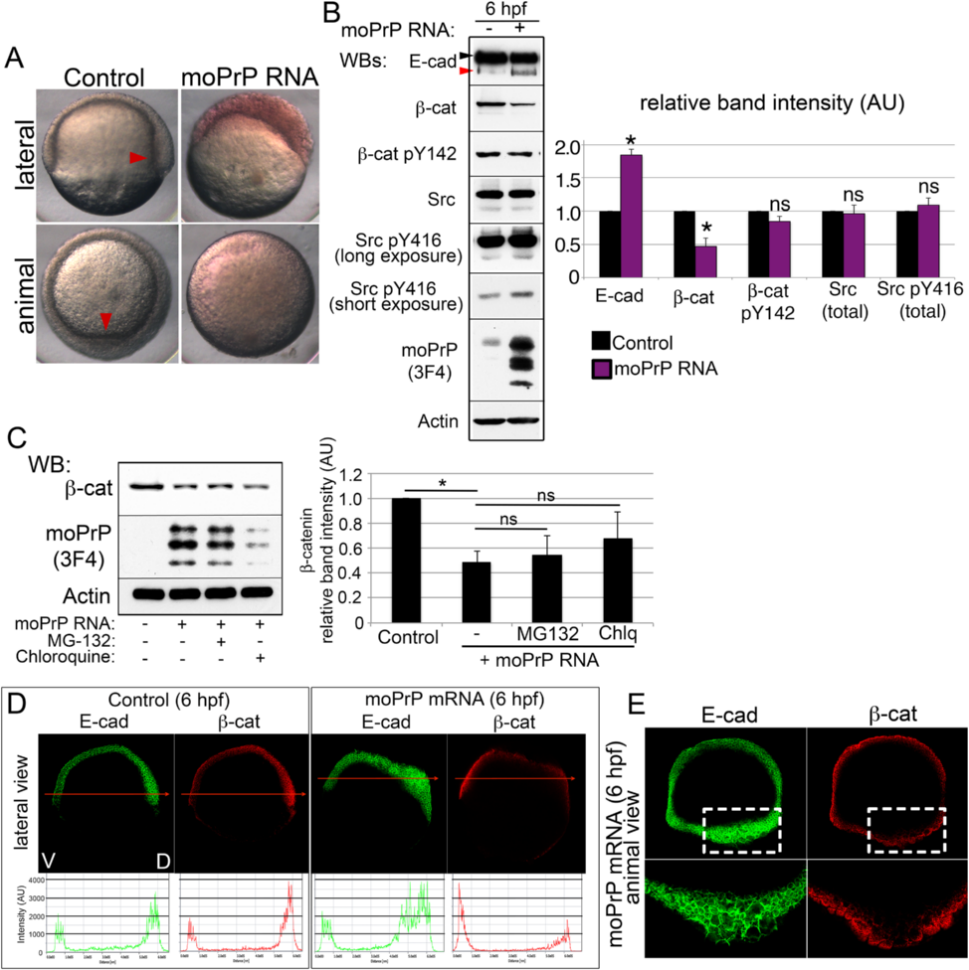
Morphological and molecular phenotypes upon PrP overexpression. **a**. Shield formation (red arrowhead) in 6 hpf embryos. See also Additional file 2: Video S1. **b**. Levels of E-cadherin, β-catenin and SFKs in 6 hpf embryos expressing mouse PrP. E-cadherin arrowheads as in Fig. 1. **c**. Effect of protein degradation inhibitors on the β-catenin levels of PrP-OE embryos. **d**. Dorsoventral distribution of AJ molecules upon PrP overexpression. Lateral views (midsections) of whole 6 hpf embryos, along with fluorescence profiles through the dorsoventral axis (indicated by red arrows; V = ventral, D = dorsal). See also Additional file 1: Figures S4 and S6. **e**. Close-up from (**d**) showing aberrant morphology of dorsal deep cells expressing mouse PrP. WB = Western blot; IF = immunofluorescence. Densitometric analysis of Western blot bands (**b** and **c**) is expressed in arbitrary units (AU); average values of four experiments ± SEM are shown; statistical significance was assessed using unpaired, two-tailed *t-*tests; ns = not significant (*p* > 0.05), * = *p* ≤ 0.05

We have reported that PrP overexpression in zebrafish induces a distinct morphological phenotype characterized by asymmetric epiboly and increased cell-cell adhesion [21, 38]. To mechanistically link the zebrafish PrP gain- and loss-of-function phenotypes, we characterized PrP-overexpressing (PrP-OE) embryos at the cellular and molecular levels. Normally, between 5 and 6 hpf, cellular structures known as the germ ring and the shield define the marginal and dorsal regions of the zebrafish gastrula, respectively [39]. PrP-OE embryos lack these morphological features and develop instead a large dorsal thickening (Fig. 5a). This abnormal accumulation of cells results in mechanical stress and bursting of the embryo before completion of epiboly (Additional file 2: Video S1). We reasoned that if PrP-1 knockdown destabilizes AJ components, PrP overexpression would enhance their cell surface expression and lead to the observed cell clumping. In fact, embryos overexpressing mouse PrP showed normal plasma membrane localization of E-cadherin and β-catenin at 6 hpf (Additional file 1: Figure S5A). Surprisingly, while their levels of mature E-cadherin were increased (Fig. 5b), those of β-catenin were strongly reduced and could not be restored with MG-132 or chloroquine (Fig. 5c). Given the asymmetric deformation of these embryos, we asked if the opposite changes in the levels of E-cadherin and β-catenin might stem from local alterations in their distribution along embryonic axes. Whole mount immunostainings of 6 hpf control embryos revealed an increasing, ventral to dorsal gradient of both proteins (fluorescence profiles in Fig. 5d). In contrast, embryos overexpressing mouse or zebrafish PrPs exhibited increased dorsal E-cadherin accumulation and reduced dorsal β-catenin (Fig. 5d and Additional file 1: Figure S5B). Concomitant with these alterations, dorsal cells appeared deformed and enlarged (Fig. 5e). Thus, cell surface E-cadherin becomes locally up-regulated in the dorsal cells of PrP-OE embryos, whereas the associated β-catenin is down-regulated via a mechanism other than protein degradation.

**Fig. 6:**
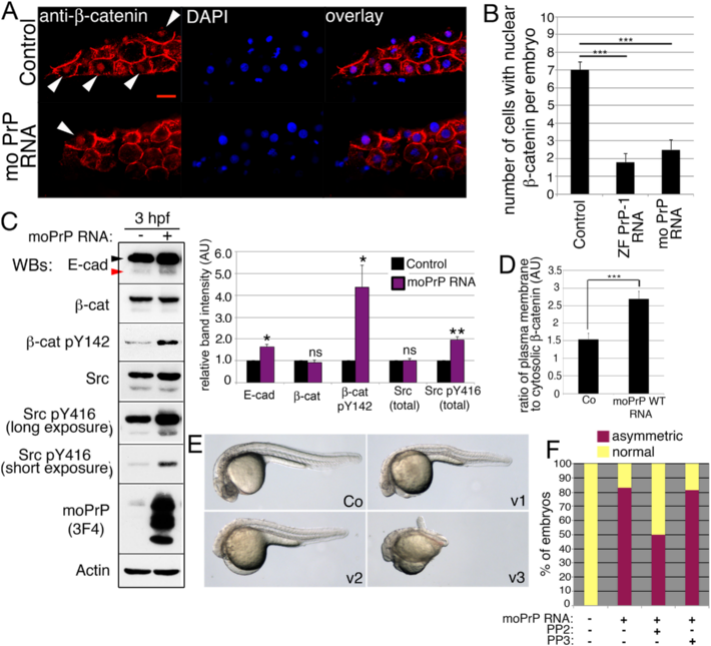
PrP overexpression up-regulates E-cadherin, inhibits nuclear β-catenin and produces ventralized embryos. **a**. Localization of β-catenin in the nuclei of dorsal marginal blastomeres at 3 hpf (immunofluorescence). Arrowheads indicate β-catenin-positive nuclei. Scale bar = 20 μm. **b**. Quantification of cells with nuclear β-catenin in 3 hpf embryos expressing zebrafish and mouse WT PrP constructs. Average numbers of cells with nuclear β-catenin per embryo ± SEM are shown (*n* = 10, three independent experiments); statistical significance was assessed using unpaired, two-tailed *t-*tests; *** = *p* ≤ 0.001. **c**. Levels of E-cadherin, β-catenin and SFKs in 3 hpf embryos expressing mouse PrP (Western blot and densitometric analysis). E-cadherin arrowheads as in Fig. 1. Densitometric analysis of Western blot bands expressed in arbitrary units (AU); average values of three independent experiments ± SEM are shown; statistical significance was assessed using unpaired, two-tailed *t-*tests; ns = not significant (*p* > 0.05), * = *p* ≤ 0.05, ** = *p* ≤ 0.01. **d**. Ratios of plasma membrane to cytosolic β-catenin immunofluorescence in dorsal blastomeres of 3 hpf embryos. Mean ratios ± SEM are from 10 cells/embryo (*n* = 7, three independent experiments); statistical significance assessed by unpaired, two-tailed *t-*test, *** = *p* < 0.001. **e**. Ventralized hypomorphic phenotypes (v1-3) of 1 dpf embryos injected with 20 ng/μl mouse PrP mRNA. **f**. Quantification of 6 hpf embryos with abnormal dorsoventral specification upon injection of mouse PrP alone, together with PP2, or with its inactive analog PP3

Crucially, β-catenin signaling is pivotal to dorsal specification. As early as the 128-cell stage (2.25 hpf), cytosolic β-catenin enters the nuclei of a small group of marginal blastomeres to activate the transcription of dorsalizing genes [40]. Given the lack of shield and reduced levels of dorsal β-catenin in 6 hpf PrP-OE embryos, we considered the possibility that PrP might disturb earlier stages of axis formation by locally depleting dorsal nuclear β-catenin. Indeed, quantitative confocal analysis of dorsal marginal blastomeres at 3 hpf showed a clear reduction in the average number of cells stained for nuclear β-catenin, from ~7 in control embryos to ~1.8 and ~2.5 in embryos overexpressing zebrafish PrP-1 or mouse PrP, respectively (Fig. 6a and b; *p* < 0.001). Therefore, the dorsalizing nuclear function of β-catenin in these early embryos is compromised as a result of increased PrP activity.

In mouse embryonic stem cells, cell surface E-cadherin can bind and sequester β-catenin at the plasma membrane, thus preventing its internalization and nuclear translocation [27]. Given the dorsally increased levels of cell surface E-cadherin in 6 hpf PrP-OE embryos, we examined if a similar mechanism could induce the observed reduction of dorsal nuclear β-catenin earlier, at 3 hpf. Fittingly, Western blot analysis of 3 hpf PrP-OE embryos revealed a ~60 % increase in the levels of mature, cell surface E-cadherin (Fig. 6c). Moreover, fluorescence measurements indicated that the ratio of plasma membrane vs. cytosolic β-catenin was 1.8 times higher in the dorsal cells of PrP-OE embryos than in their control counterparts (Fig. 6d). This difference was not due to cytosolic degradation of β-catenin, as control and PrP-OE embryos showed comparable levels of this protein at 3 hpf (Fig. 6c). Rather, the increased plasma membrane localization suggests that β-catenin is retained by cell surface E-cadherin, thus reducing the pool of cytosolic molecules available for translocation to the nucleus.

It is well-established that preventing the nuclear localization of β-catenin in dorsal blastomeres produces ventralized embryos by 1 dpf [41]. This effect could only rarely be observed in PrP-OE embryos because they died from excessive cell adhesion before completing gastrulation. We circumvented this problem by microinjecting lower mRNA amounts to overexpress PrP at sub-lethal levels. The resulting PrP hypomorphic embryos completed epiboly and by 1 dpf presented a distribution of ventralized phenotypes with variable penetrance and expressivity (Fig. 6e). The anterior and dorsal defects varied in strength, ranging from mildly reduced eyes, brain and notochord to completely undifferentiated heads and shortened anterior-posterior axes. This result further supports the notion that PrP overexpression impairs the role of β-catenin in axis formation by exacerbating its adhesive function.

Finally we asked if, consistent with the PrP-1 knockdown data, SFKs also mediate the PrP gain-of-function phenotype. In fact, at 3 hpf, the relative levels of activated (p-Tyr416) Src/Yes and Fyn were elevated in PrP-OE embryos (Fig. 6c). This higher SFK activity was further evident from the corresponding increase in the levels of p-Tyr142-β-catenin (Fig. 6c). Moreover, blocking SFK activity with the pharmacological inhibitor PP2 reduced the proportion of 6 hpf PrP-OE embryos with abnormal morphology from 83.26 % to 49.97 % (Fig. 6f; *p* < 0.01). To ascertain whether this early increase in SFK activity may contribute to the dorsal accumulation of cell surface E-cadherin, we analyzed their distribution along embryonic axes. Confocal analyses of whole untreated embryos at 3 hpf revealed that SFKs and E-cadherin are similarly distributed in a ventral to dorsal gradient (Additional file 1: Figure S5C). Interestingly, this gradient was more pronounced in PrP-OE embryos, suggesting that exogenous PrP enhances E-cadherin adhesion preferentially in dorsal blastomeres by activating their already higher levels of SFKs. Of note, the conserved link between PrP and SFK activation was also observed in human MCF-7 cells, where expression of mouse or fish PrPs triggered the accumulation of SFKs at contacts sites and the corresponding increase in SFK activity (Additional file 1: Figure S6). Altogether, our data point at the positive regulation of SFK activity and AJ function by PrP as the underlying mechanism behind the zebrafish PrP gain- and loss-of-function phenotypes.

### Functionality of mouse PrP mutants in zebrafish embryos

**Fig. 7:**
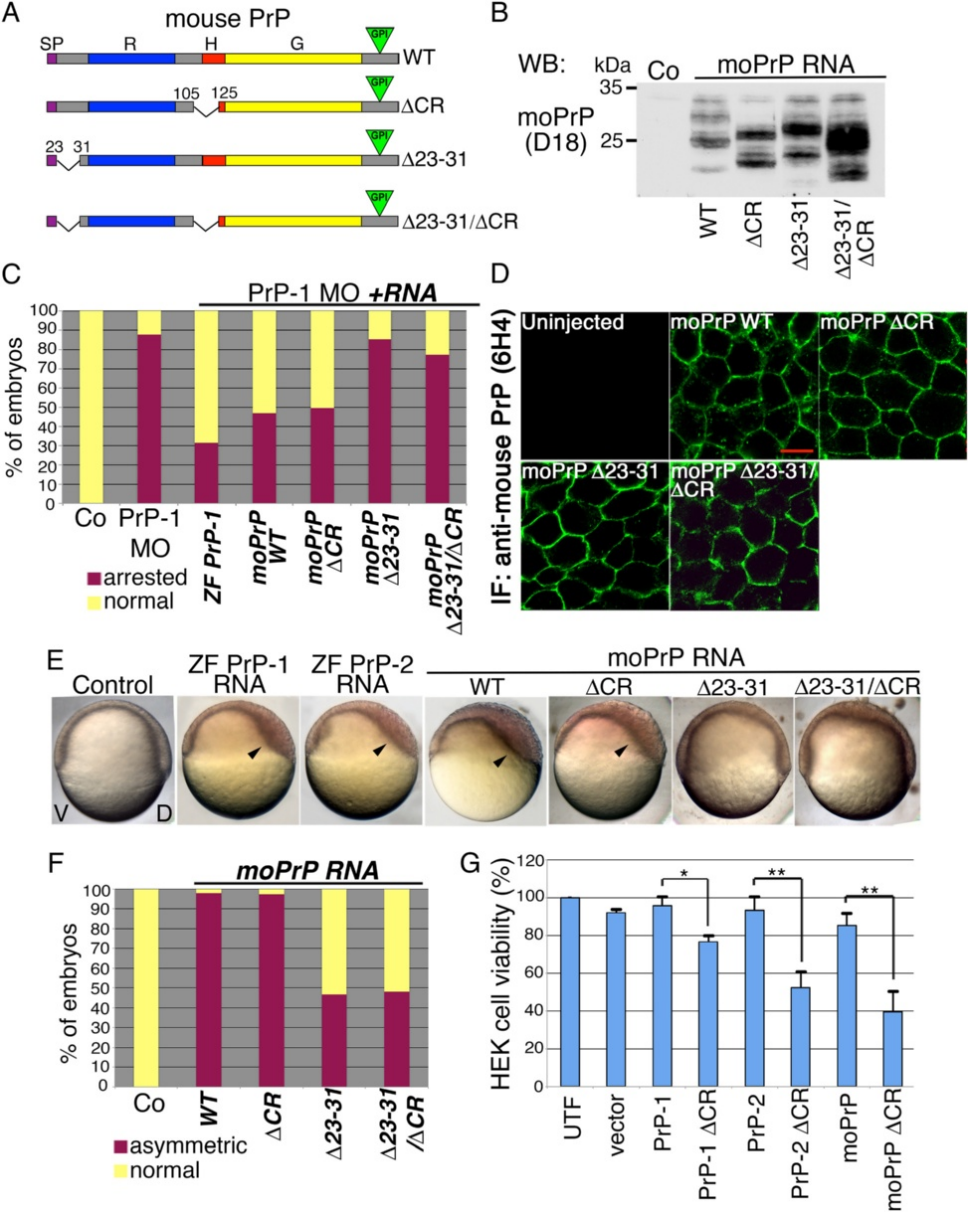
Effect of Δ23-31 and ΔCR deletions on PrP activity. **a**. Mouse PrP deletion mutants. Protein domains are marked with different colors: SP = signal peptide, R = repetitive domain, H = hydrophobic stretch, G = globular domain; green triangles = GPI-anchor. **b**. Detection of mouse WT or mutant PrPs using the D18 anti-PrP antibody on 6 hpf embryo protein lysates. **c**. Phenotypic quantification of 7 hpf embryos co-injected with PrP-1 morpholinos and PrP mRNAs. **d**. Localization of mouse PrP constructs in the deep cells of 6 hpf embryos (6H4 anti-PrP antibody staining). Scale bar = 10 μm. **e**. Embryonic phenotypes upon expression of zebrafish (ZF) or mouse (mo) PrP mRNAs (7 hpf, lateral views); arrowheads show abnormal dorsal thickening; V = ventral, D = dorsal. **f**. Quantification of 7 hpf embryos with dorsoventral phenotypes upon expression of mouse PrPs. Mean values are shown in (**c**) and (**f**). **g**. MTT viability assay of HEK cells expressing mouse or zebrafish PrPΔCRs after Zeocin treatment. WB = Western blot; IF = immunofluorescence. Data are shown as the average percentage of A_570_ values in untreated cells ± SEM; statistical significance was assessed using unpaired, two-tailed *t-*tests, * = *p* ≤ 0.05, ** = *p* ≤ 0.01

Deletions in the central region (ΔCR, residues 105-125) and the N-terminal polybasic stretch (Δ23-31) of mouse PrP (Fig. 7a) modulate its neurotoxic activities. For instance, PrPΔCR triggers a lethal neurodegenerative phenotype in transgenic mice [42] whereas PrPΔ23-31 abrogates this neurotoxic effect as well as the rescuing (neuroprotective) activity of WT PrP [43–45]. Because mouse PrP can partially revert the zebrafish PrP-1 knockdown phenotype [21, 38], we used mRNA rescue experiments to assess whether the ΔCR and Δ23-31 mutations modify the conserved PrP function required for zebrafish epiboly. After verifying and titrating expression of the mouse constructs in zebrafish embryos (Fig. 7b), mRNAs and PrP-1 morpholinos were co-injected into fertilized eggs, and the resulting phenotypes were scored at 6 hpf (Fig. 7c). Notably, PrPΔCR retained full activity and rescued the PrP-1 morphants as efficiently as WT PrP. In contrast, PrPΔ23-31 showed a strongly reduced rescuing activity (~74 % relative to WT PrP), similar to an additional mutant carrying both deletions, PrPΔCR/Δ23-31. The reduced activity of the Δ23-31-contanining constructs was not due to deficient cell surface expression, as demonstrated by whole-mount immunofluorescence (Fig. 7d).

To confirm the rescue data without the confounding effect of the PrP-1 knockdown phenotype, we also conducted gain-of-function experiments and measured the ability of the mutants to induce the conserved PrP overexpression phenotype (Fig. 7e). In these assays, the activity of the ΔCR construct was again comparable to that of WT PrP (~95 % of embryos with overexpression phenotype), whereas deletion of residues 23-31 reduced this effect by ~50 % (Fig. 7f). Importantly, these results reflect the ability of the constructs to inhibit nuclear β-catenin signaling (Additional file 1: Figure S7), as expression of WT and ΔCR PrPs significantly reduced the numbers of marginal blastomeres positive for nuclear β-catenin (2.5 and 3.7 cells/embryo, respectively, vs. 7 cells/untreated embryo). Moreover, β-catenin nuclear translocation was barely affected by overexpression of Δ23-31 or Δ23-31/ΔCR PrPs (7.8 and 7.7 cells/embryo, respectively). Therefore, we conclude that the N-terminal polybasic stretch (residues 23-31) is critical for the control of embryonic cell adhesion by PrP, and that the ΔCR mutation does not significantly impair this PrP function.

Importantly, expression of mouse PrPΔCR in the presence or absence of endogenous PrP-1 did not cause enhanced toxicity or lethality in fish embryos. A possible explanation is that ΔCR toxicity requires the expression of a developmentally regulated PrP partner absent from the zebrafish gastrula. Alternatively, ΔCR toxicity might be a specific property of mammalian PrPs. To address this issue, we asked whether zebrafish PrPΔCRs can induce toxicity in an established mammalian cell model. For this, we generated ΔCR versions of zebrafish PrP-1 and -2 (Additional file 1: Figure S4), and tested them in HEK cells using the drug-based cellular assay (DBCA) [46]; changes in cell viability were assessed by the MTT assay. Notably, cells expressing either zebrafish or mouse PrPΔCRs were less viable than those expressing the corresponding WT PrPs (Fig. 7g; 20 %, 40 % and 45 % reduction in MTT metabolic activity for PrP-1-, PrP-2- and mouse PrP-expressing cells; *p* < 0.05, *p* = 0.01, and *p* = 0.01, respectively). This suggests that the role of the central region as a suppressor of cytotoxicity is evolutionarily conserved and that the corresponding toxic cascade in zebrafish may become active only after gastrulation.

### PrP-dependent influence of Aβ oligomers on zebrafish embryonic SFKs and AJs

**Fig. 8:**
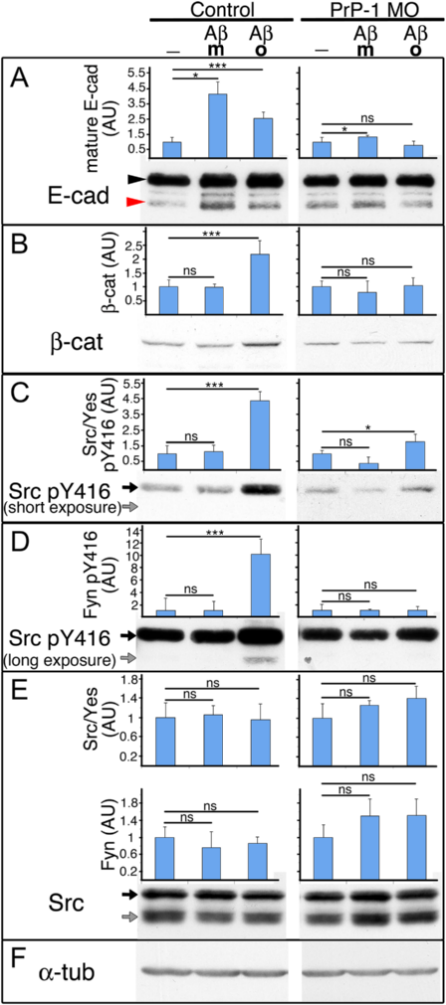
Aβ oligomers induce a PrP gain-of-function effect in zebrafish embryonic cells. Levels of AJ proteins (**a** and **b**) and SFKs (**c**-**e**) in 6 hpf embryonic cells upon treatment with Aβ monomers (m) or oligomers (o). Actin was used as a protein loading control (**f**). E-cadherin arrowheads as in Fig. 1; arrows = distinct SFKs bands (black: Src/Yes; grey: Fyn). Densitometric analysis of Western blot bands is shown in arbitrary units (AU). Data were collected from three independent experiments (average values ± SEM); statistical significance was assessed using unpaired, two-tailed *t-*tests, * = *p* ≤ 0.05, *** = *p* ≤ 0.001

In mammalian neurons, binding of Aβ oligomers to neuronal PrP activates Fyn and blocks the endocytosis of the NR2B subunit of the NMDA receptor (NMDAR), leading to glutamate excitotoxicity and synaptic damage [7]. In our zebrafish embryos, PrP-mediated activation of Fyn and Yes prevents the endocytosis of E-cadherin to support embryonic cell adhesion. The obvious similarity between these two unrelated events prompted us to ask if Aβ oligomers would modulate zebrafish embryonic SFKs and AJ components in a PrP-dependent manner. As a negative control, we used monomeric Aβ, which unlike oligomeric Aβ, does not induce Fyn activation (Um et al, 2012). To facilitate access of Aβ to embryonic cell surfaces, we dissociated 5 hpf zebrafish control and PrP-1 morphant embryonic cells and allowed them to re-aggregate for one hour in the presence or absence of 0.5 μM Aβ oligomers before analyzing them for changes in the levels of SFKs and AJ components. Notably, Aβ oligomers triggered increased levels of p-Tyr416 (activated) Src/Yes and Fyn (4.4-fold and 10.1-fold increase, respectively; *p* < 0.001) without significantly affecting their total levels (Fig. 8c-e). In addition, the levels of E-cadherin and β-catenin were elevated by 2.6- and 2.2-fold, respectively (*p* < 0.001, Fig. 8a and b). The increase in p-Tyr416 SFKs and β-catenin was dependent on endogenous PrP-1 expression and Aβ oligomerization, since it was observed neither in PrP-1 morphant cells nor upon exposure to Aβ monomers. Interestingly, the increase in mature E-cadherin was also PrP-1-dependent but it was indistinctively triggered by Aβ monomers or oligomers (see Discussion). Thus, in zebrafish blastocytes, Aβ oligomers induce biochemical changes consistent with a PrP gain-of-function scenario.

## Discussion

Identifying conserved molecular pathways controlled by PrP^C^ in vivo is an important step towards understanding mechanisms of neurodegeneration. Using multiple knockdown and cross-rescue experiments, we define here a genetic pathway in which SFKs act downstream of PrP-1 to support embryonic AJ function. Although Fyn activation by PrP is widely recognized, its physiological significance had so far remained unclear. This notion can now be extended to Yes, which we here identify as a previously unknown effector of PrP in vivo. As with our findings in zebrafish, synergistic contributions of SFKs to cell adhesion are evident in Fyn/Src and Fyn/Yes knockout mice [47]. Furthermore, single Fyn, Yes or Src knockouts exhibit mild developmental phenotypes but double (Fyn/Src or Src/Yes) and triple knockouts are embryonic lethal [48, 49]. The specific requirement of SFKs during gastrulation is particularly evident in frogs and zebrafish [30, 50 and this study]. Aside from these similarities across model organisms, differences are also apparent: Fyn/Yes knockout mice undergo kidney degeneration but remain viable [49], whereas Src is essential for epiboly in *Xenopus* but not in zebrafish [30, 50]. Our data on PrP, Fyn and Yes suggest that PrP utilizes multiple SFKs to modulate a wide spectrum of cellular responses. Hence, the functional redundancy of SFKs and the diversity of their downstream targets might explain the differences between the PrP loss-of-function phenotypes of mice and zebrafish [13].

Both our gain- and loss-of-function data indicate that PrP-1 positively regulates Fyn and Yes activity. Does this imply that PrP can directly activate SFKs? To our knowledge, PrP has neither kinase nor phosphatase activities. Moreover, it is not clear whether the GPI-anchor of PrP can extend across the plasma membrane and interact with cytoplasmic SFKs. Nevertheless, clustering of PrP at cell-cell contacts can induce coalescence and cross-activation of SFK molecules, or their activation by PrP-associated, tissue-specific trans-membrane proteins like N-CAM or mGluR5 [29, 51]. In addition, the GPI-anchor of PrP influences its interaction with signaling complexes by determining its localization within distinct membrane microdomains [52]. Particularly relevant to our findings is the modulation of SFKs through the preferential degradation of their activated (p-Tyr416) forms via lysosomal pathways [53]. In fact, activated SFKs were reduced in PrP-1 morphants and this effect partially required lysosomal function. Moreover, it was recently shown that the degradation of activated SFKs can switch from proteasomal to autophagic/lysosomal when a key stimulus such as focal adhesion kinase (FAK)-signaling is absent [54]. Similarly, the loss of PrP-1 could direct activated SFKs to a lysosomal degradation pathway. Thus, PrP-1 supports SFK function either by inducing their activation and/or by eliciting a signal that inhibits the degradation of their activated forms.

Because PrP enhances the levels and plasma membrane localization of E-cadherin and β-catenin via SFKs, our data also offer mechanistic insights into poorly understood aspects of AJ regulation. For instance, while classical studies in oncogene-transformed cells found that SFK activity disrupts AJs, more recent reports indicate the opposite. This contradiction has been explained through a “bimodal” model in which SFKs promote cell adhesion at low, physiological levels but suppress it at high, oncogenic levels [33]. The positive regulation of AJs by SFKs in zebrafish (this study) and *Drosophila* embryos [55] provides in vivo support for this model. On the other hand, it is widely recognized that Tyr phosphorylation of β-catenin by SFKs promotes AJ disassembly [56]. How could the same phosphorylation events have positive *and* negative effects on AJs? In our analyses, β-catenin Tyr142 was indeed phosphorylated by SFKs but increased pTyr142 levels did not result in the loss of cell adhesion. Therefore, similar to [47, 57], our experiments suggest that Tyr phosphorylation of β-catenin is not the predominant event leading to the disruption of AJs under physiological conditions.

Importantly, SFKs also phosphorylate E-cadherin and modify its accessibility to binding partners that compete to promote or prevent its retention at the plasma membrane. Of key relevance among these molecules are AP-2 -a clathrin-associated mediator of endocytosis- and p120, which protects E-cadherin from AP-2 binding and Hakai-mediated ubiquitination [58]. Our finding that SFK activity prevents the clathrin-dependent endocytosis and degradation of E-cadherin is consistent with this mechanism and suggests that the regulation of E-cadherin endocytosis overrides the effect of β-catenin (Tyr) phosphorylation on AJ stability. Along the same lines, blocking E-cadherin endocytosis in PrP-1 morphants restored the plasma membrane association of β-catenin, whereas preventing β-catenin degradation did not rescue E-cadherin levels. These data further suggest that increased E-cadherin endocytosis precedes and drives β-catenin internalization upon PrP-1 knockdown. Since key regulators of AJ turnover like AP-2, Hakai, p120 and protein tyrosine phosphatases (PTPs) are themselves targets of SFKs, more complex regulatory scenarios cannot be excluded at this time.

Consistent with our PrP-1 knockdown data, PrP overexpression induced a lethal gastrulation phenotype by enhancing SFK activity and E-cadherin cell surface expression. This accounts for the increased Ca^+2^-dependent aggregation observed in PrP-OE embryos [21], as well as for their morphogenetic defects and death by mechanical disruption. Unexpectedly, β-catenin is not up-regulated along with E-cadherin in these embryos but instead its early role as a dorsalizing signal becomes lessened. Its increased cell surface localization and reduced levels in mid-blastula dorsal nuclei agree with the reported ability of E-cadherin to sequester β-catenin at the plasma membrane [27]. A similar phenomenon is known in *Xenopus* and *Drosophila* embryos, where E-cadherin up-regulation antagonizes β-catenin signaling [59–61]. Interestingly, β-catenin can promote its own transcription [62, 63], suggesting that its reduction in the dorsal nuclei of 3 hpf PrP-OE embryos might impair its local biosynthesis. Accordingly, dorsal β-catenin is down-regulated by 6 hpf and this effect is not due to protein degradation. Although no specific antibodies are available to distinguish between the two zebrafish β-catenins (β-catenin-1 and -2), our data suggest that PrP modifies the function of at least β-catenin-2, as only this duplicate has dorsalizing activity [64].

Canonical Wnts promote β-catenin signaling by preventing the degradation of its cytosolic pool [56]. In contrast, PrP overexpression inhibits β-catenin signaling by stabilizing it at the plasma membrane. This might suggest that PrP antagonizes Wnts in dorsal marginal cells of the blastula. However, the two pathways likely act independently because β-catenin degradation is neither increased in PrP-OE embryos nor reduced in PrP-1 morphants. In addition, mutants of the zebrafish dorsalizing gene Wnt8 exhibit a different dorsoventral phenotype with no defects in cell adhesion [65]. Interestingly, a modulatory effect of PrP on Wnt signaling may depend on its sub-cellular localization. For instance, in the nucleus of intestinal epithelial cells, PrP can interact with β-catenin and promote the transcription of Wnt-target genes [66]. Such a mechanism was not evident in our overexpression experiments and may therefore be restricted to differentiated cell types with significant levels of PrP and β-catenin in the nucleus. Given the prominent role of Wnt signals in cancer and neurodegeneration, it will be of interest to explore additional regulatory interactions between PrP and Wnt across cell types and tissues.

The ability of mouse PrP to modulate SFKs and AJs in the zebrafish gastrula is greatly enhanced by residues 23-31, which also control neuronal survival and neurotoxicity in transgenic mice [43–45]. Could such dissimilar activities of PrP have a common regulatory basis? The 23-31 equivalent regions of mouse and zebrafish have low amino acid sequence identity. However, their conserved basic character suggests that positive charge is key to their function. In fact, N-terminal polybasic regions can greatly enhance the plasma membrane association of many proteins via electrostatic interactions with membrane lipids. As demonstrated for Gα subunits, the ensuing lateral segregation within plasma membrane sub-regions has a profound effect on the molecule’s signaling properties [67]. Along these lines, we have proposed that the polybasic N-terminus of PrP allows its insertion into the plasma membrane [43]. Therefore, the 23-31 region of PrP could indirectly facilitate its interaction with distinct functional partners located at special microdomains of the plasma membrane.

PrPΔCR neurotoxicity in mice has been attributed to a disrupted interaction between the CR and a hypothetical neuroprotective partner [42]. The effect of zebrafish PrPΔCRs on HEK cell viability (this study) implies that this phenomenon is conserved across vertebrate PrPs. Yet, no ΔCR-associated lethality was evident in zebrafish gastrulae, suggesting that the relevant cytotoxic molecule is absent in early embryonic cells. Accordingly, PrPΔCR causes toxicity in neurons only upon exposure to glutamate or cationic drugs [68]. Moreover, ΔCR transgenic mice become ill only one week after birth [42]. Therefore, a related effect in fish may be restricted to neuronal cell types at later developmental stages and/or require induction by additional factors.

The WT-like activity of the ΔCR construct in our assays shows that the CR is not essential for embryonic PrP function. Nevertheless, the ability of PrPΔCR to induce spontaneous ionic currents in cultured cells [69] adds to increasing evidence showing that PrP can influence ion channel function at neuronal plasma membranes [70, 71]. Given the known role of SFKs as modulators of synaptic ion channel activity [13, 72], the PrP/SFK pathway described here provides a testable molecular hypothesis linking PrP dysfunction, abnormal SFK activity and downstream alterations in synaptic physiology.

Remarkably, nanomolar concentrations of human Aβ oligomers activate the PrP/SFK cascade both in mammalian neurons [7] and zebrafish embryonic cells (this study), leading to increased NMDAR or AJ stability at the plasma membrane, respectively. The similarity between these findings extends beyond the common involvement of PrP and SFKs. Although structurally and functionally unrelated, NR2B and E-cadherin are membrane proteins whose endocytic trafficking is negatively modulated by Tyr phosphorylation. The existence of a molecular mechanism able to control both cell adhesion and synaptic transmission has interesting implications for neurodegeneration, as cadherin adhesive complexes play critical roles in synapse formation and plasticity [73], and their inhibition accelerates Aβ-induced synaptic damage [74]. Our data suggest that PrP/SFK-mediated dysregulation of cadherins and NMDARs jointly contribute to Aβ-induced synaptic failure. This is an interesting scenario that can now be experimentally addressed in Alzheimer’s research models.

In line with the study by Um and colleagues [7], we did not observe SFK activation by Aβ monomers. The reason for this might be stoichiometric: an Aβ oligomer -containing up to 100 monomeres [75]- is likely to bind more PrP molecules than a monomer and induce the amount of PrP clustering required for SFK activation. Nonetheless, Aβ monomers did up-regulate E-cadherin in an SFK-independent manner, suggesting that Aβ monomers and oligomers activate mutually exclusive pathways that converge in the modulation of E-cadherin expression. Among the molecules that could mediate the effect of Aβ monomers on E-cadherin, receptor tyrosine kinases (RTKs) are particularly interesting. Many RTKs localize to AJs; some -like the epidermal growth factor receptor (EGFR)- can phosphorylate cadherin/catenin complexes independently of SFKs [76] and interact with PrP [77]. Moreover, the insulin-like growth factor-1 receptor (IGF-1R) mediates a neuroprotective activity of Aβ monomers [78], binds and modulates E-cadherin [79], and is expressed in the zebrafish gastrula [80]. Therefore, our results support the view that Aβ monomers exert biological activities distinct from those of Aβ oligomers [81]. The ability of the Aβ peptide to interact with any given partner (PrP or RTKs) probably depends on its aggregation state, as oligomerization causes its C-terminal half to assume a unique conformation that masks some of its amino acid side chains and creates new epitopes from its polypeptide backbone [82–84]. On the other hand, the biochemical changes induced by human Aβ peptides in zebrafish cells strongly suggest that the Aβ-binding sites of PrP are evolutionarily conserved structures. In fact, zebrafish PrPs contain positively charged regions similar to those identified as the two major Aβ-binding sites of mammalian PrPs [75, 85].

Altogether, our work lays out a mechanistic framework in a simple model organism to explore the physiological complexity of PrP-associated signals, their connection to protein aggregation diseases, and their potential use in the identification of novel therapeutic targets and compounds.

## Conclusions

The present study combines genetic, biochemical and cell biological approaches in zebrafish embryos to explore the physiological significance of PrP signaling and expand our knowledge about the regulation of SFKs and AJ components during gastrulation. In particular, we show that: **1)** PrP activates not only Fyn but also the related SFK member Yes, and that it preferentially controls the degradation of their activated forms; **2)** Fyn and Yes positively regulate E-cadherin by preventing its endocytosis and degradation; **3)** Tyrosine phosphorylation of β-catenin is not the predominant event leading to AJ disruption; **4)** PrP-mediated control of the SFK/AJ pathway requires the same N-terminal stretch needed for its neuroprotective activity; **5)** Exposure to Aβ oligomers overactivates the SFK/AJ pathway in a PrP-dependent manner.

This early developmental mechanism is also relevant to the study of PrP pathogenesis, as it provides us with a simple experimental system to monitor the cellular events impaired by mammalian neurotoxic PrP mutants in vivo. The fact that Aβ oligomers trigger PrP-dependent changes not only in Fyn but also in Yes function suggests that multiple SFKs and their diverse cellular targets are common players in prion and Alzheimer’s pathologies. Furthermore, our observations relate to key oncogenic pathways because they illustrate how SFKs regulate AJs under physiological conditions. On the other hand, the similar control of E-cadherin and NMDAR endocytosis via PrP- and SFK-dependent tyrosine phosphorylation suggests a general role of PrP in controlling the stability of membrane proteins in diverse physiological scenarios. Altogether, our findings highlight the zebrafish as a powerful tool to dissect the complex roles of PrP in physiology and disease as well as to facilitate drug discovery efforts.

## Methods

### Zebrafish

Wild-type (WT) zebrafish were kept at the University of Konstanz's animal facility following standard procedures [86]. Embryos were raised and staged as described previously [21].

### Molecular cloning

Plasmids encoding zebrafish WT *Fyn* and *Yes* cDNAs, a kind gift of Dr. Jeroen den Hertog, were used to create C-terminally tagged EGFP fusion constructs. The following PCR primers (Eurofins MWG Operon) were designed to remove stop codons: Fyn-F-(EcoRI): 5′-CGAATTCATGGGCTGCGTACAGTG-3′ and Fyn-R-(ApaI): 5′-GGGGCCCAGAGGTTGTCCCCGGGTTGG-3′; Yes-F-(EcoRI): 5′-CGAATTCATGGGCTGCGTAAAAAGC-3′ and Yes-R-(ApaI): 5′-GGGGCCCACAGGTTGTCTCCGGGCTGATA-3′. Constitutively active (CA) forms were generated by mutating Tyr residues 531 in Fyn and 540 in Yes to Phe using instead the following reverse primers: Fyn CA-R-(ApaI): 5′-GGGGCCCAGAGGTTGTCCCCGGGTTGGAAC-3′ and Yes CA-R-(ApaI): 5′-GGGGCCCACAGGTTGTCTCCGGGCTGAAAC-3′. PCR products were cloned into pCRII-TOPO (Invitrogen), digested with EcoRI/ApaI and subcloned into pEGFP-N1 (Clontech). For expression in zebrafish embryos, inserts lacking 9 bp upstream of the EGFP stop codon were excised with EcoRI/BsrGI, and inserted into the corresponding sites of pCS2 + -EGFP, thereby restoring the full-length fusion constructs. pCS2 + -EGFP was constructed by inserting EGFP into the EcoRI/XbaI sites of pCS2+. Mouse PrP cDNAs containing the 3 F4 epitope were excised from pcDNA3 and inserted into pCS2+ at the following sites: HindIII/BamHI for WT, ΔCR and Δ23-31/ΔCR PrP, and HindIII/XhoI for Δ23-31 PrP. The missing 92 bp fragment of pCS2+ downstream of the HindIII site (containing the SP6 promoter) was re-introduced using the cloning oligos SalI/HindIII-oligo 1: 5′-TCGACgatttaggtgacactatagaatacaagctacttgttctttttgcaagctaaatccactgtgatatcttatgttcgatgaacaagaaaaacgtTTCGA-3′ and SalI/HindIII-oligo-2: 5′-AGCTTtgcaaaaagaacaagtagcttgtattctatagtgtcacctaaatcG-3′ (Eurofins MWG Operon). Oligos were annealed for 10 min at 50 °C and the resulting fragment was inserted into pCS2+ at the SalI/HindIII sites. Zebrafish ΔCR PrPs were generated by deleting amino acids 374-393 (GFGKKAVVAAGVGAMAGMAI) in PrP-1 and 295-314 (GFGKQAIIAAGAGAVAGMAL) in PrP-2 via inverse PCR. The corresponding WT cDNAs in pCS2+ were used as templates with the following primer combinations: PrP-1-ΔCR-Fwd: 5′- pGGCTATGGAATAGGAAACTTTCAACG -3′/PrP-1-ΔCR-Rev: 5′- pTTTGGATTTTGCAGAAGGGTTGTAGC -3′, and PrP-2-ΔCR-Fwd: 5′- pGGATATGGCCTGGGAAGTTTCCCCCG -3′/PrP-2-ΔCR-Rev: 5′- pCTTTGATTTGTAAGAAGGGGCCATAC -3′. PCR products were blunt end ligated to generate circular DNA.

### Morpholino and mRNA injections

Lissamine-tagged, non-overlapping morpholinos against *PrP-1* were described earlier [21]: MO-PrP1-1: 5′-GGTCCATAAAAAGGTTGAAGAAGCG-3′ and MO-PrP1-2: 5′-TCTCTCCCGCAGCACTCTCTGCTCA-3′. Similarly designed morpholinos against zebrafish *Fyn* and *Yes* [30] were purchased from Gene Tools (Oregon): Fyn: 5′-TGTCCTTACATTGCACACAGCCCAT-3′ and Yes: 5′-CCTCTTTACTCTTGACACAGCCCAT-3′. Morpholinos were microinjected at 0.5 ng/nl (PrP-1-1 and -2, each) and 1.6 ng/nl (Fyn and/or Yes, each) in 1X Danieau buffer/0.125 % Phenol Red (Sigma). Capped mRNAs were synthesized in vitro using the mMessage mMachine SP6 kit (Ambion). For comparable levels of PrP overexpression, mRNAs were microinjected in 0.05 M KCl/0.125 % Phenol Red at 40 ng/μl (WT PrP), 30 ng/μl (ΔCR and Δ23-31 PrP) and 20 ng/μl (Δ23-31/ΔCR PrP and PrP-1). For PrP rescue or hypomorph experiments, mRNAs were co-injected at half the above concentrations. For Fyn and Yes rescue experiments, mRNAs were co-injected with morpholinos at 11 pg/nl (WT) and 1 pg/nl (CA), each. Microinjections were carried out at the one- to-four-cell stage using an injection volume of 5 nl per embryo. For phenotypic analyses, at least 200 embryos per experiment were examined in three independent experiments. Average numbers of embryos with a given phenotype are given as the percentage of total embryos treated, and statistically validated with two-tailed unpaired t-tests. Images of live embryos were acquired with a LUMAR.V12 microscope (Zeiss) and processed using Axiovision and Adobe Photoshop CS5.

### Chemical inhibitors

Pharmacological inhibitors were co-injected with morpholinos or mRNAs at 100 μM (chloroquine, Sigma), 50 mM (ammonium chloride, Calbiochem), 10 μM (MG-132, Calbiochem) in 0.05 % DMSO; 10 or 50 μM (Dynasore, Calbiochem) in 0.1 % or 0.02 % DMSO, respectively; and 5 μM (PP2 or PP3) in 0.05 % DMSO; all final concentrations.

### Immunofluorescence

Embryos were stained as described previously [21]. The following primary antibodies were used: mouse monoclonal anti E-cadherin (BD Biosciences, 1:250), anti β-catenin (BD Biosciences, 1:250), anti-phosphotyrosine (Cell Signaling, 1:500), anti-PrP 6H4 (Prionics, Switzerland); rabbit polyclonal anti β-catenin (Sigma, 1:500) and anti-Src (Cell Signaling, diluted 1:100). Secondary antibodies: Alexa-488 conjugated goat anti-rabbit or -mouse and Cy3-conjugated donkey anti-rabbit or -mouse (Jackson Immunoresearch, 1:1000), Cy5 conjugated goat anti-rabbit or -mouse (Invitrogen, 1:1000). Whole embryos were imaged as flat (deyolked) or thick (with yolk) mounts on LSM 510 and 710 confocal microscopes (Zeiss), and images were further processed using Adobe Photoshop CS5. Differences in protein distribution along embryonic axes were visualized using fluorescence profiles generated with LSM 510 and ZEN software (Zeiss). To study β-catenin translocation, Z-sections of whole embryos were generated and marginal cells with nuclear β-catenin were counted. To determine ratios of plasma membrane vs. cytosolic β-catenin in dorsal blastomeres, whole cell/cytoplasm areas were outlined and the corresponding fluorescence (integrated densities) measured and subtracted using Image J.

### Western blot

Embryos were dechorionated in 1 mg/ml Pronase (Sigma) for 10 min at 30 °C, washed four times in 30 % Danieau buffer, and mechanically dissociated in deyolking and wash buffer as previously described [87]. The dissociated embryonic cells were disrupted in ice-cold lysis buffer (20 mM Tris–HCl, pH 7.5, 2 mM EDTA, 100 mM NaCl, 5 mM MgCl_2_, 1 % Triton X-100, 10 % glycerol) supplemented with 1X Halt Protease and Phosphatase Inhibitor Cocktail (Thermo Scientific). Protein concentrations were determined using the Bradford Assay (Sigma), and 17 μg of lysate per lane (approximately ten 6-hpf embryos) were loaded onto 10 % or 12 % SDS gels. For improved detection of E-cadherin isoforms, embryos were dissociated on ice and directly lysed in SDS loading buffer. The following primary antibodies were diluted in TBS-0.1 % Tween containing 3 % bovine serum albumin (Sigma Aldrich): mouse monoclonal anti-E-cadherin (BD Biosciences, 1:1000), anti- β-catenin (BD Biosciences, 1:1000), Prion (3F4) antibody (Covance); rabbit polyclonal anti-phospho-β-catenin Y142 (1:500), anti-δ-catenin (1:1000), anti-α-tubulin (1:5000) from Abcam, and anti-Src (1:1000), anti-phospho-Src Y416 (1:500), anti-phospho-Src Y527 (1:500) from Cell Signaling; goat polyclonal anti-Actin (Santa Cruz Biotechnology, 1:1000). The specificity of commercial antibodies on total and phosphorylated zebrafish proteins was carefully assessed through comparison of fish and mammalian amino acid target epitopes, preliminary Western blots and immunofluorescence controls, as well as experiments published by us and colleagues [21, 34, 38]. Secondary, HRP-conjugated antibodies: rabbit polyclonal goat anti-rabbit or rabbit anti-goat IgG (H + L) (Jackson Immunoresearch, 1:10000); goat polyclonal anti-mouse IgG (H + L) (Invitrogen, 1:5000) and anti-human IgG (Sigma Aldrich, 1:10000). The anti-PrP hybridoma D18 was provided by Dennis R. Burton (The Scripps Research Institute, La Jolla, CA); the purified antibody was used at 0.5 μg/ml. The intensity of Western blot signals was quantified using ImageJ and statistically evaluated using Excel and Graphpad Prism 6.0d.

### Drug-based cellular assay (DBCA)

HEK293 (ATCC CRL-1573) cell culture conditions, the DBCA, and MTT assay have been described previously [46]. Cells were plated at 70 % confluency, transfected for 6 h with Lipofectamine 2000 (Invitrogen), and further incubated for 18 h before treatment with 0.5 mg/ml Zeocin for 24 h at 37 °C.

### Aβ treatment

Embryos were dissociated at 5 hpf as described above and cells were incubated for 1 h at 28.5 °C in 30 % Danieau medium containing 0.5 μM monomeric or oligomeric Aβ. Aβ oligomers were prepared as reported previously [88]. Cell lyses and WB analyses were performed as described above.

## Additional files

Additional file 1: Figure S1. Recovery of epiboly in PrP-1 morphants by treatment with degradation and endocytosis inhibitors. A. Treatment of PrP-1 morphants with DMSO alone does not restore epiboly. B. Treatment with protein degradation inhibitors restores AJ components at the plasma membrane of PrP-1 morphants (only MG-132 shown). Immunofluorescence (IF) analysis of E-cadherin and β-catenin in the deep cell layer of 6 hpf embryos. Scale bar = 10 μM. **Figure S2.** Fyn and Yes act downstream of PrP-1 during epiboly. A. Immunofluorescence (IF) of E-cadherin and β-catenin in 6 hpf Fyn and Yes single knockdown embryos. Scale bar = 10 μM. B. Photos of live embryos at 6 hpf displaying normal or arrested epiboly. Recovery of epiboly is visible in PrP-1 morphants expressing EGFP-tagged WT or CA Fyn and Yes. Lissamine (Lis)-tagged PrP-1 morpholinos were used. **Figure S3.** Changes in levels of total or phosphorylated SFKs, and phospho-tyrosine upon PrP-1 knockdown. A. SFK levels in 6 hpf embryos (WB) after injection with increasing PrP-1 morpholino doses. B. Changes in relative p-Tyr527 levels in PrP-1 morphants, as measured by densitometric analysis of WB bands. WB for total or p-Tyr527 SFKs was performed with 6 hpf embryo extracts. Values for phospho Y527 SFKs were normalized to those of total SFKs. Average values ± SEM of three independent experiments are depicted. C. Immunofluorescence of total phosphorylated tyrosine in deep and EVL cells in 6 hpf embryos. Scale bar = 10 μM. **Figure S4.** Sequence comparison of mouse and zebrafish N-terminal polybasic and central regions (CR). A. Basic residues are marked in turquoise and gray boxes indicate repetitive domains. B. Boxed areas indicate the CRs. Hydrophobic domains are marked in red. **Figure S5.** Effects of embryonic PrP OE on the localization of SFKs and AJ components. A. Immunofluorescence in dorsal deep cells of 6 hpf embryos (animal views); arrowheads point at plasma membrane localization; scale bar = 10 μM. B. Gastrula midsections of 6 hpf ZF PrP-1 overexpressing embryos, immunostained as indicated and shown from lateral or animal perspectives. Graphs present fluorescence profiles along the ventral-dorsal axis (indicated by red arrows; V = ventral, D = dorsal). C. Blastula midsections of 3 hpf embryos (two different planes from animal view), immunostained as indicated; arrowheads point at areas of increased immunofluorescence. **Figure S6.** Changes in Src activation and localization upon expression of mouse and zebrafish PrPs in MCF-7 cells. A. WB analysis with lysates of MCF-7 cells expressing EGFP-tagged PrPs. B. Subcellular distribution of EGFP-PrPs and Src immunofluorescence in MCF-7 cells. Scale bar = 10 μM. C. Quantification of Src immunofluorescence at cell-cell contact sites. Graph depicts percentages of cells (average values ± SEM) with different percentage of Src at cell-cell contact sites. Values were normalized to whole cell Src immunofluorescence. **Figure S7.** Quantification of cells with nuclear β-catenin in 3 hpf embryos expressing various PrP constructs. Average numbers of cells with nuclear β-catenin per embryo ± SEM are shown (*n* = 10, three independent experiments). (DOCX 3704 kb) Additional file 2:
**Video S1.** PrP OE induces a lethal gastrulation phenotype characterized by mechanical disruption. Time-lapse recording between 6 and 9 hpf. Control embryo (left), PrP-1 morphant embryo (center), and embryo overexpressing mouse PrP (right). (MOV 11369 kb)
