# Supplementary material for: Activation of zebrafish Src family kinases by the prion protein is an amyloid-β-sensitive signal that prevents the endocytosis and degradation of E-cadherin/β-catenin complexes in vivo
Source: Mol Neurodegener. 2016 Feb 9;11:18. doi: 10.1186/s13024-016-0076-5 (PMC4748561; doi:10.1186/s13024-016-0076-5)

**Additional File**

**Additional file summary:** the file contains Figures S1 to S7 and Video S1

**Figure S1**


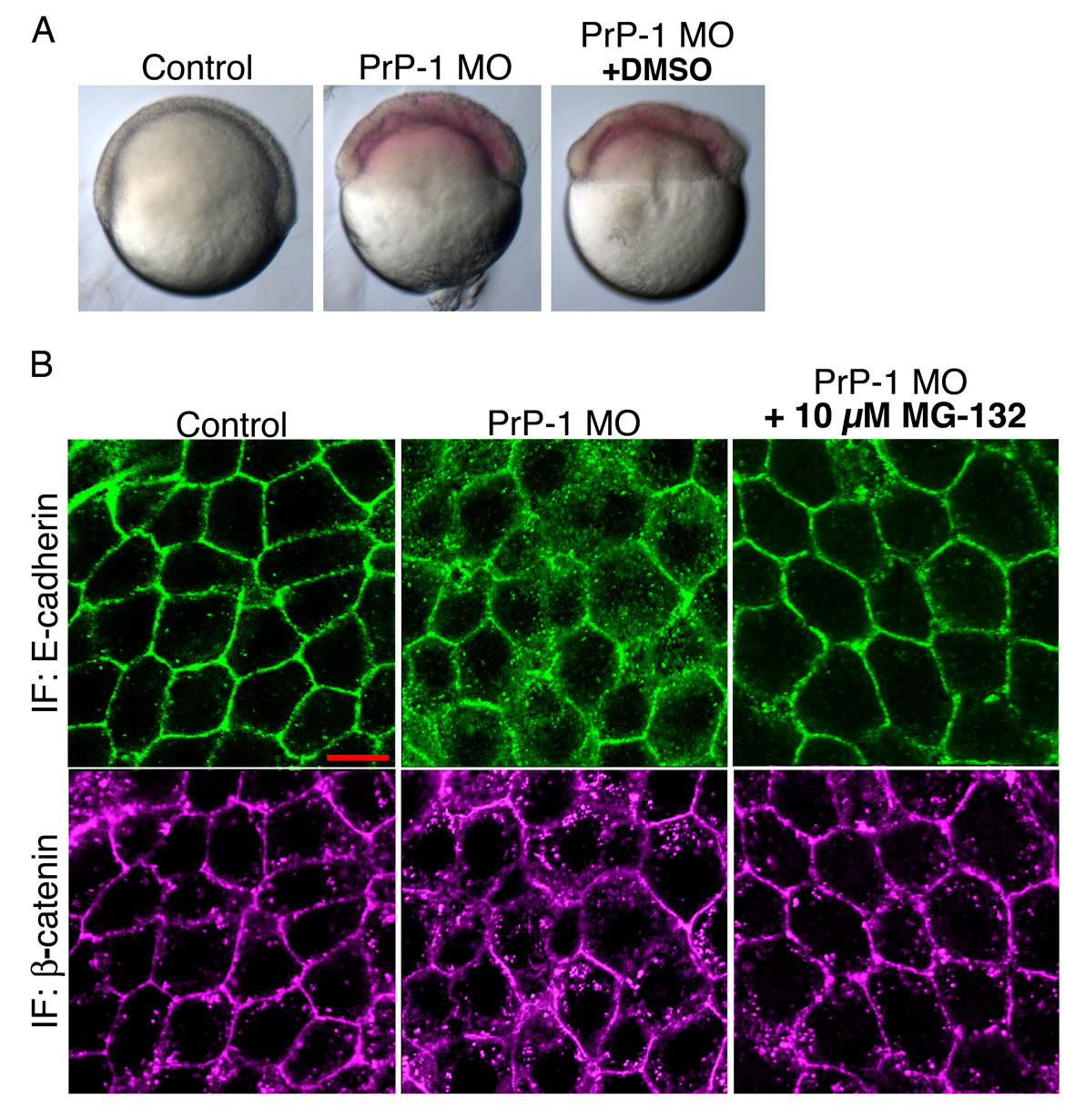


**Figure S2**


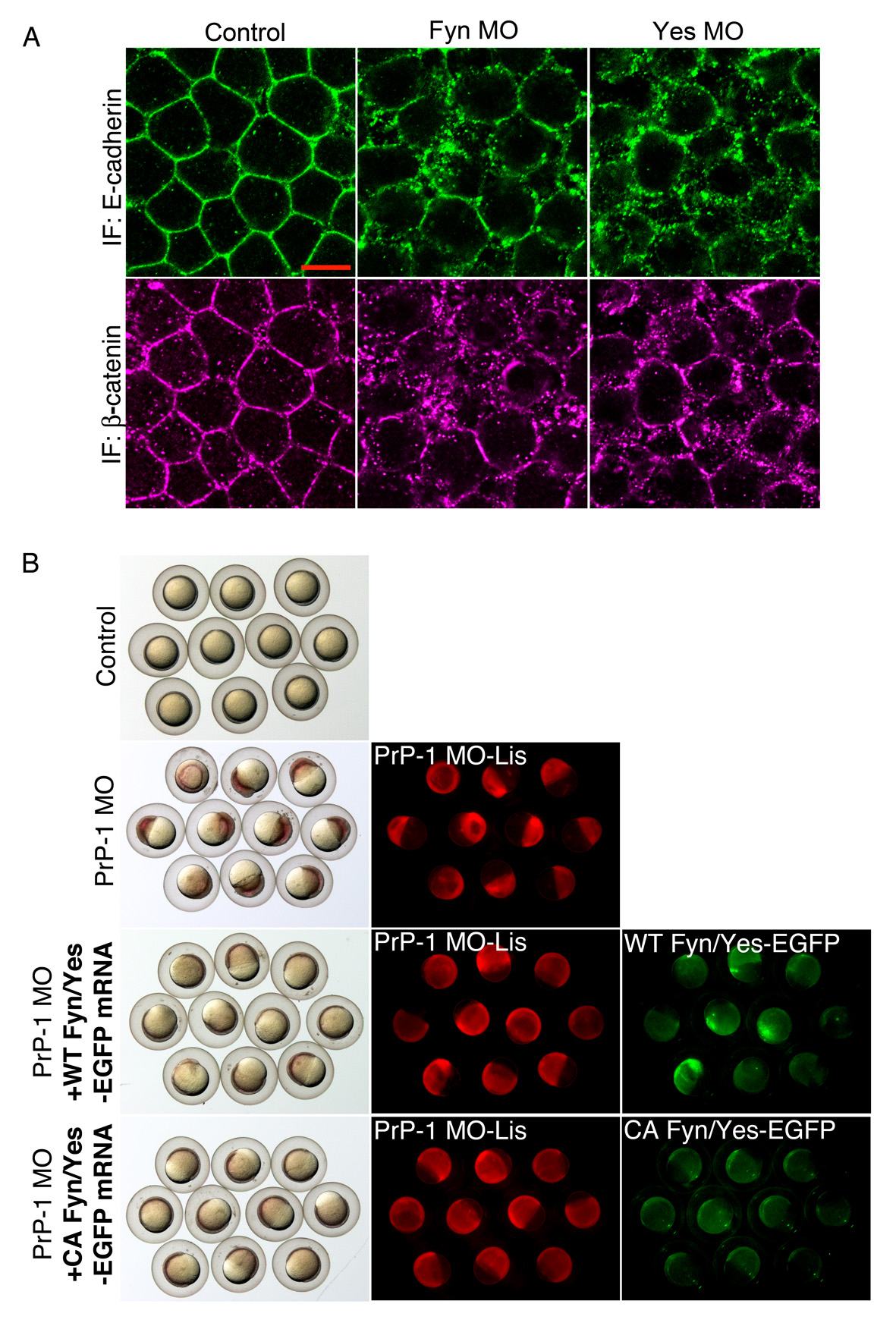


**Figure S3**


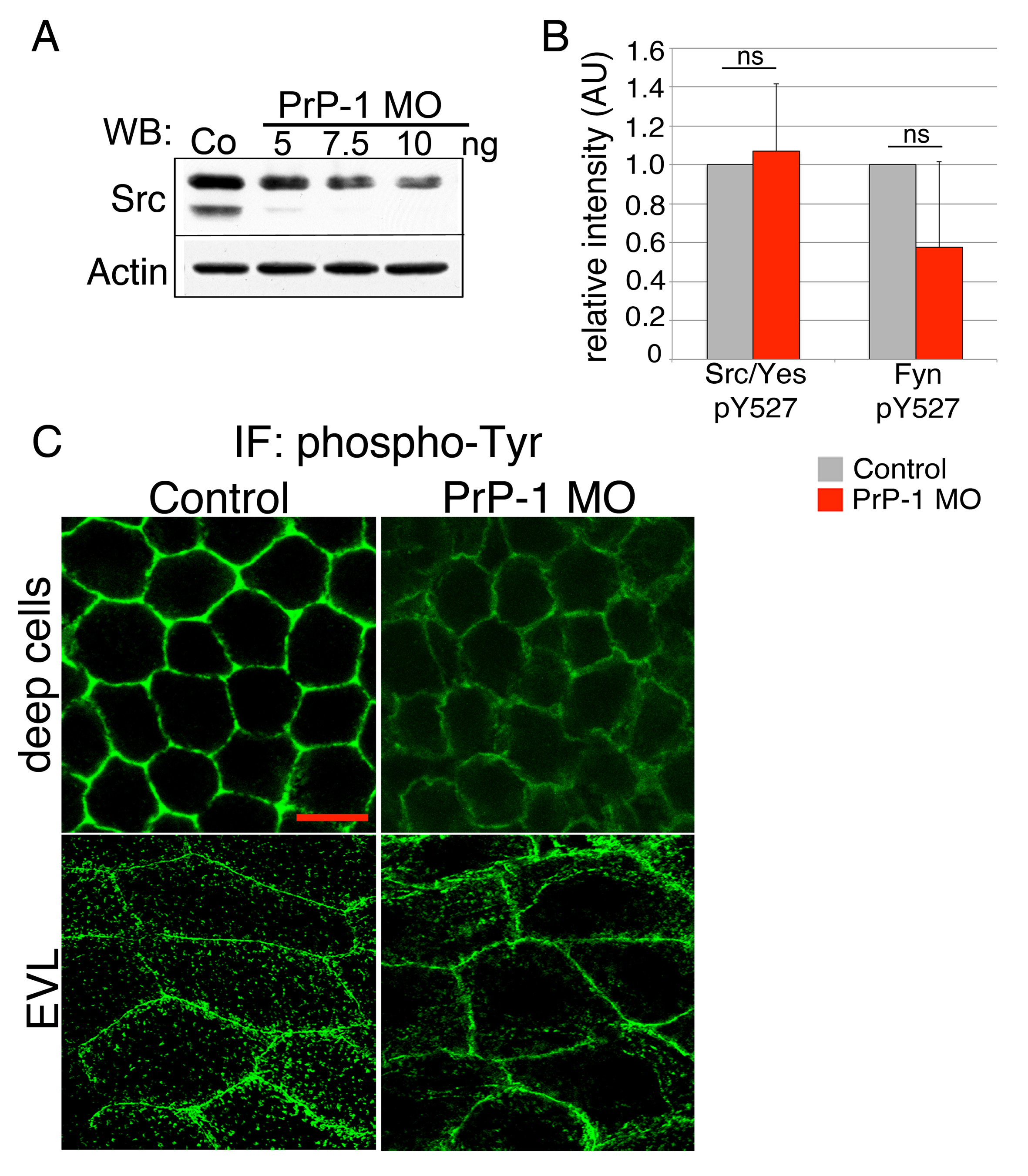


**Figure S4**


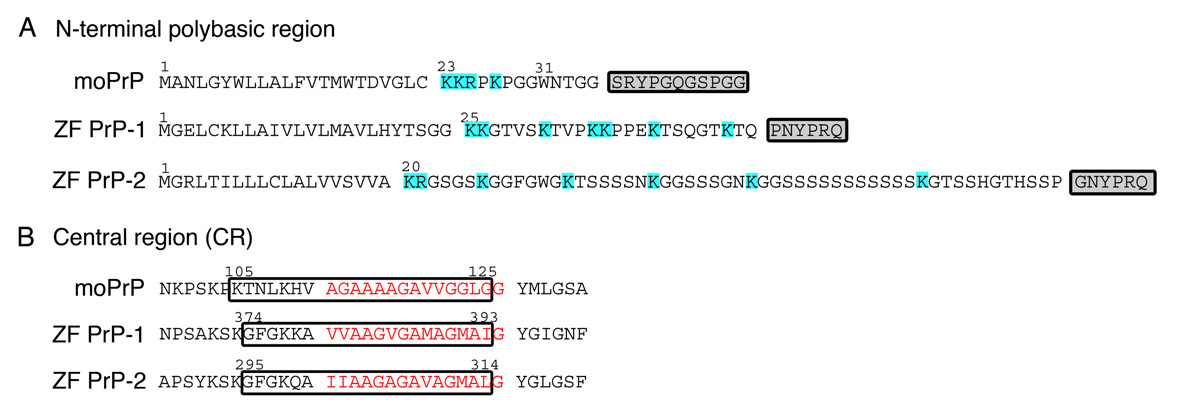


**Figure S5**


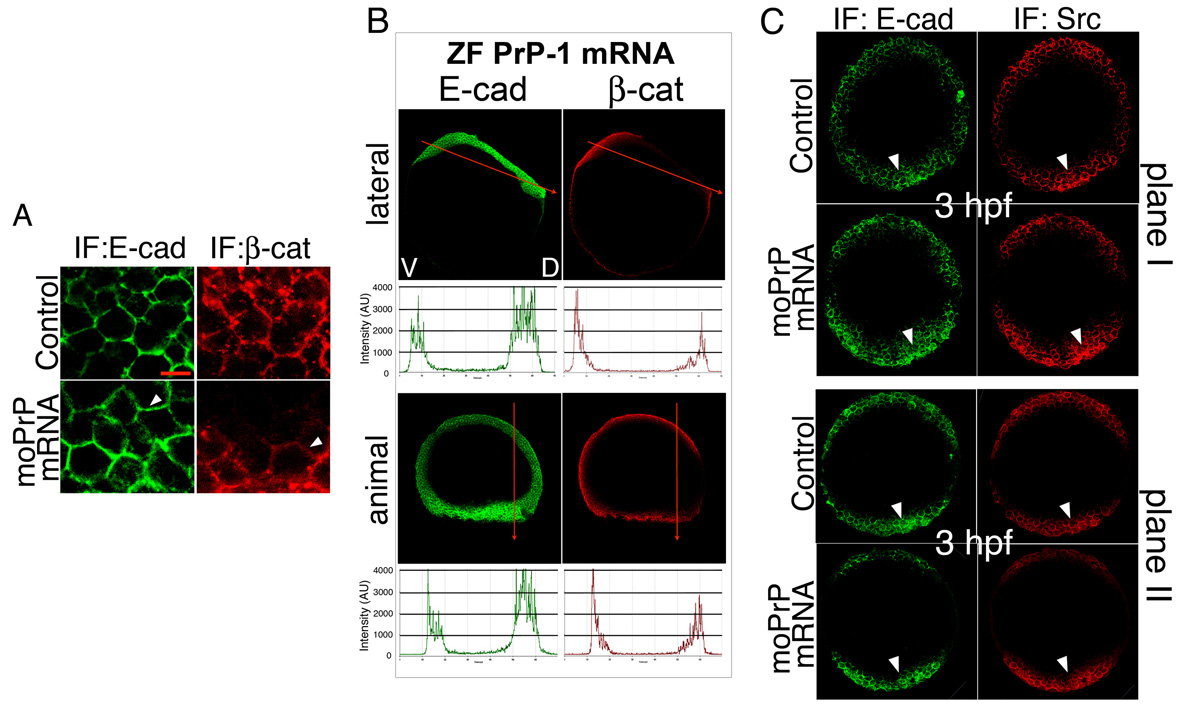


**Figure S6**


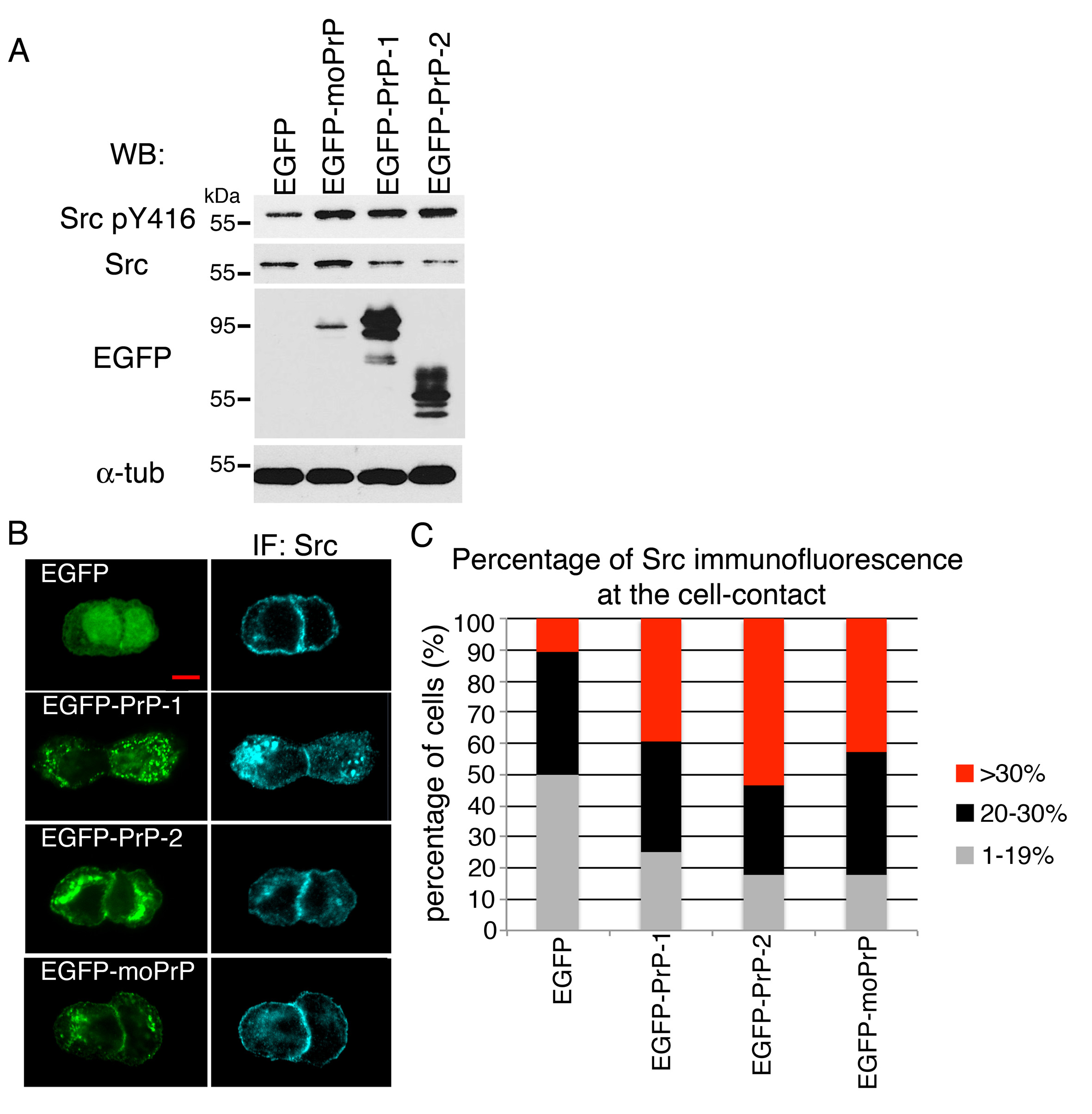


**Figure S7**


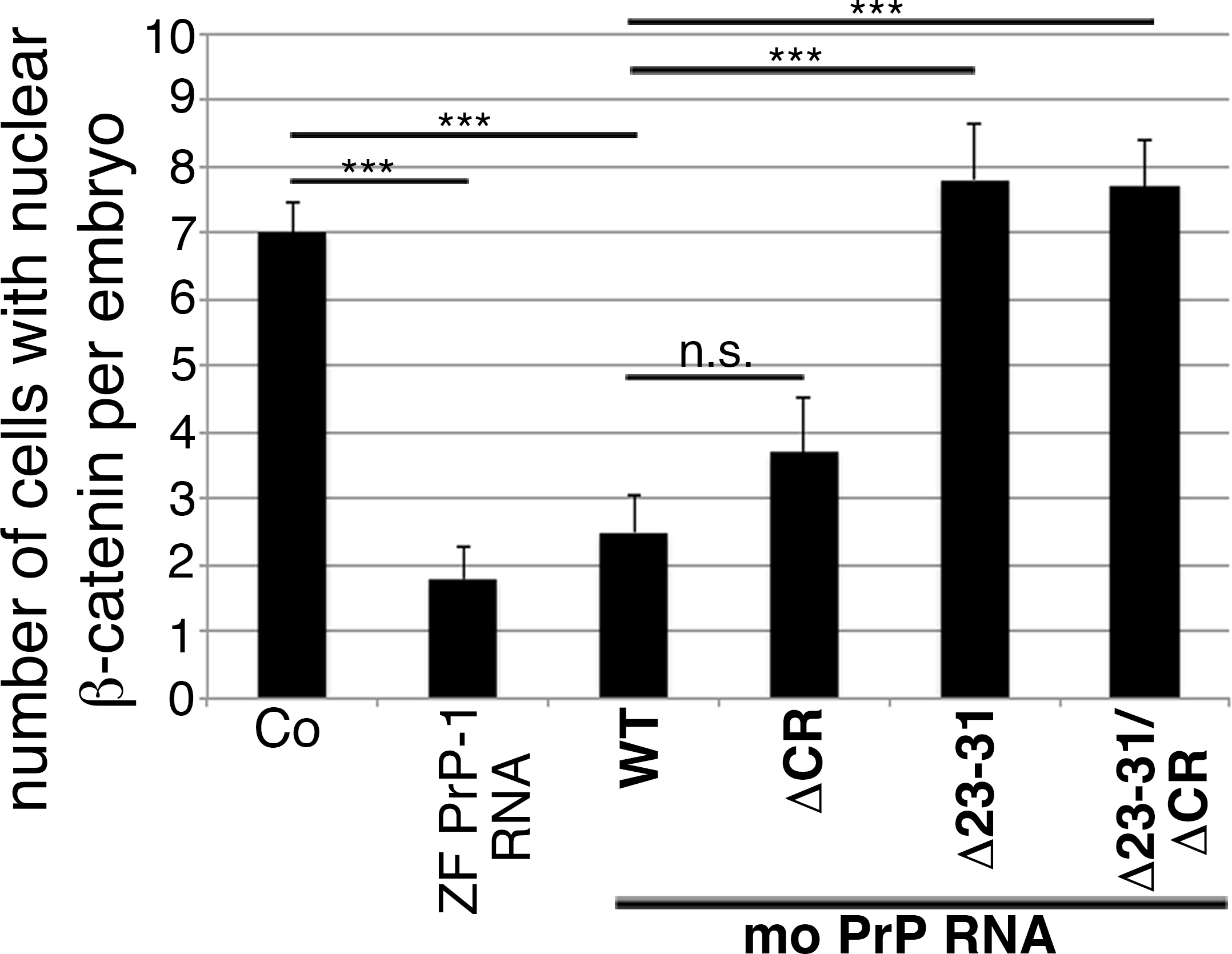

Supplement: Additional file 1: Figure S1. — Recovery of epiboly in PrP-1 morphants by treatment with degradation and endocytosis inhibitors. A. Treatment of PrP-1 morphants with DMSO alone does not restore epiboly. B. Treatment with protein degradation inhibitors restores AJ components at the plasma membrane of PrP-1 morphants (only MG-132 shown). Immunofluorescence (IF) analysis of E-cadherin and β-catenin in the deep cell layer of 6 hpf embryos. Scale bar = 10 μM. Figure S2. Fyn and Yes act downstream of PrP-1 during epiboly. A. Immunofluorescence (IF) of E-cadherin and β-catenin in 6 hpf Fyn and Yes single knockdown embryos. Scale bar = 10 μM. B. Photos of live embryos at 6 hpf displaying normal or arrested epiboly. Recovery of epiboly is visible in PrP-1 morphants expressing EGFP-tagged WT or CA Fyn and Yes. Lissamine (Lis)-tagged PrP-1 morpholinos were used. Figure S3. Changes in levels of total or phosphorylated SFKs, and phospho-tyrosine upon PrP-1 knockdown. A. SFK levels in 6 hpf embryos (WB) after injection with increasing PrP-1 morpholino doses. B. Changes in relative p-Tyr527 levels in PrP-1 morphants, as measured by densitometric analysis of WB bands. WB for total or p-Tyr527 SFKs was performed with 6 hpf embryo extracts. Values for phospho Y527 SFKs were normalized to those of total SFKs. Average values ± SEM of three independent experiments are depicted. C. Immunofluorescence of total phosphorylated tyrosine in deep and EVL cells in 6 hpf embryos. Scale bar = 10 μM. Figure S4. Sequence comparison of mouse and zebrafish N-terminal polybasic and central regions (CR). A. Basic residues are marked in turquoise and gray boxes indicate repetitive domains. B. Boxed areas indicate the CRs. Hydrophobic domains are marked in red. Figure S5. Effects of embryonic PrP OE on the localization of SFKs and AJ components. A. Immunofluorescence in dorsal deep cells of 6 hpf embryos (animal views); arrowheads point at plasma membrane localization; scale bar = 10 μM. B. Gastrula midsections of 6 hpf ZF PrP-1 overex [file 13024_2016_76_MOESM1_ESM.docx]
